# ZBP1 in Neuroinflammation and Neurodegeneration: Z-Nucleic-Acid Sensing, RHIM Signalling and Therapeutic Targeting

**DOI:** 10.3390/ijms27167478

**Published:** 2026-08-21

**Authors:** Matei Șerban, Corneliu Toader, Răzvan-Adrian Covache-Busuioc

**Affiliations:** 1 Faculty of General Medicine, “Carol Davila” University of Medicine and Pharmacy, 050474 Bucharest, Romania; mateiserban@innbn.com (M.Ș.);; 2 Department of Neurosurgery, “Carol Davila” University of Medicine and Pharmacy, 050474 Bucharest, Romania; 3 Department of Vascular Neurosurgery, National Institute of Neurology and Neurovascular Diseases, 077160 Bucharest, Romania; 4 Puls Med Association, 051885 Bucharest, Romania

**Keywords:** ZBP1, neuroinflammation, Alzheimer’s disease, innate immunity, Z-nucleic acids, necroptosis, ADAR1, mitochondrial DNA

## Abstract

In contrast to foreign nucleic acids, some of our own endogenously synthesized nucleic acids may become immunologically active without being considered “foreign”. For example, abnormalities in chromatin organization, transcription termination, ribonucleic acid (RNA) splicing, and RNA editing, together with damage to mitochondrial integrity, may render normally functional deoxyribonucleic acid (DNA) and RNA persistently available and aberrantly structured ligands for innate immunity. Z-DNA-binding protein 1 (ZBP1), recently identified as an important component of this innate immune system, recognizes both left-handed DNA (Z-DNA) and left-handed RNA (Z-RNA) using its tandem Z-alpha (Zα) domains and couples recognition of these conformational states to receptor-interacting serine/threonine-protein kinase 1 (RIPK1)-, receptor-interacting serine/threonine-protein kinase 3 (RIPK3)-, and mixed-lineage kinase domain-like pseudokinase (MLKL)-dependent inflammatory and cell-death pathways. More recent studies have also shown that ZBP1 plays a role in recognizing damaged self-nucleic acids associated with tauopathies, Alzheimer’s disease (AD), traumatic brain injury (TBI), and amyloid-associated neuroinflammation. The nucleic-acid forms associated with these conditions include transposable-element activation, extended repeat-containing transcripts, RNA–RNA duplexes or RNA:DNA hybrids, oxidized mitochondrial DNA (mtDNA), and intercellularly transferred nucleic acids, all of which may exhibit substrate structures compatible with Z-form formation. Signaling by ZBP1 does not occur simply based upon nucleic-acid abundance; rather, signaling occurs after prolonged exposure to a nucleic acid when it persists in a structurally competent state, sufficient receptors are present to bind its exposed regions, the receptor proteoforms are competent to participate in signaling, receptor-interacting protein homotypic interaction motif (RHIM)-dependent assembly occurs, and the appropriate adaptor molecules are present. Furthermore, the identity of the cell type expressing ZBP1 determines whether the response produces RIPK3–MLKL-dependent neuronal injury, microglia-mediated inflammation, apoptosis, or mixed cell death. Finally, competition with adenosine deaminase acting on RNA 1 (ADAR1), melanoma differentiation-associated protein 5 (MDA5), double-stranded RNA-dependent protein kinase (PKR), the cyclic guanosine monophosphate–adenosine monophosphate synthase–stimulator of interferon genes (cGAS–STING) pathway, and other nucleic-acid-sensing proteins divides the available pool of endogenous nucleic acids among the outcomes of immune tolerance, type I interferon (IFN-I) signaling, translational inhibition, neuroinflammation, and necroptosis.

## 1. Introduction: Conformation as a Molecular Determinant of Nucleic-Acid Identity

### 1.1. Molecular Determinants of Ligand Identity

The identity of an endogenously produced nucleic acid is determined by several factors, including: (1) the number and arrangement of the nucleotides comprising the molecule, (2) the presence of specific modifications to individual nucleotides, (3) the presence of proteins bound to the nucleic acid, (4) its subcellular compartmentalization, (5) its duplex architecture, and (6) the overall three-dimensional shape of the molecule [1]. Noncanonical forms of nucleic acids, such as Z-DNA and Z-RNA, serve as examples of nucleic acids whose identities extend beyond sequence alone. While B-DNA and A-RNA tend to adopt right-handed helices, both Z-DNA and Z-RNA assume left-handed helical configurations. Additionally, both Z-DNA and Z-RNA contain alternating syn and anti-glycosidic conformations and a zigzag phosphodiester backbone. Several elements contribute to the ability of nucleic acids to adopt a Z-form [2]. These include: (1) the nucleotide sequence contained within the nucleic acid, (2) torsional stress, (3) negative supercoiling, (4) the presence of chemically modified bases, (5) oxidative damage to bases, and (6) interactions with specific proteins that stabilize or facilitate adoption of the Z-conformation. However, the propensity of a nucleic acid to adopt a Z-conformation does not necessarily equate to immunogenic potential [3]. Additional elements required for productive immune recognition of a structurally aberrant nucleic acid include: (1) persistence of the structural aberrancy, (2) sufficient receptor accessibility, (3) appropriate compartmentalization, and (4) sufficient ligand availability to permit downstream signaling events [4].

These considerations are especially pertinent to mammalian proteins that possess Zα domains. For example, Z-DNA-binding protein 1 (ZBP1) contains two tandem Zα domains at its amino terminus followed by receptor-interacting protein homotypic interaction motifs (RHIMs). Therefore, ZBP1 acts as a bridge between the recognition of structurally aberrant nucleic acids and inflammatory and programmed cell-death pathways [5]. Another protein that recognizes Z-conformation-compatible nucleic acids is interferon-inducible ADAR1 p150. This protein contains an N-terminal Zα domain, three double-stranded RNA-binding domains, and a C-terminal adenosine-to-inosine deaminase domain. Thus, ADAR1 p150 enables selected endogenous RNA duplexes to be recognized and edited or otherwise occluded from access by competing innate immune sensors [6]. As such, structurally similar substrates may generate different biological effects depending on the protein that interacts with them, the subcellular compartment in which the interaction occurs, and whether the interaction results in tolerance, editing, occlusion, inflammation, or cell death [7].

Therefore, we examine in this review what dictates whether an otherwise normal endogenous nucleic acid can effectively engage ZBP1. Currently available evidence suggests that multiple molecular hurdles must be overcome before productive interaction between an endogenous nucleic acid and ZBP1 can occur, rather than this process depending on a single ligand–receptor interaction [8]. First, some form of cellular perturbation must generate or expose an acceptable candidate substrate; second, the substrate must acquire a conformation compatible with interaction with a Zα domain; third, the substrate must escape sequestration by chromatin, ribonucleoproteins, or related barriers; fourth, the substrate must reside within a subcellular compartment containing accessible ZBP1; and fifth, the substrate must successfully engage a signaling-competent ZBP1 proteoform at sufficiently high local density and for a duration adequate to permit effective downstream assembly [5].

Each of these hurdles distinguishes theoretical Z-form propensity from either a transient structural intermediate or a confirmed endogenous ZBP1 ligand. We utilize the term “conformational proofreading” to denote our proposed conceptual framework describing how sequence organization and chemical history influence structural stability, compartmentalization, competitive occupancy, receptor availability, and ultimately ligand competence [9]. This term does not signify enzymatic catalysis. Rather, each barrier constrains successful activation. Oxidized DNA may become accessible without adopting a receptor-compatible structure; repetitive RNA may remain sequestered within ribonucleoprotein complexes; and Z-RNA may be captured by ADAR1 before participating in signaling-competent ZBP1 complexes. Immunogenicity may therefore arise from the convergence of structural characteristics, spatial positioning, kinetic parameters, competitive conditions, and sufficient ligand availability to support signaling [10].

Evidence supporting this model continues to grow in the context of neurological disorders. In experimental tauopathy, disruption of chromatin repression has been shown to promote transposable-element activation, accumulation of Z-RNA in neurons, and subsequent ZBP1-dependent RIPK3–MLKL signaling. Partial reduction in murine Zbp1 resulted in reduced neuronal loss, glial activation, and cognitive impairment in aged mice. Additionally, elevated levels of neuronal ZBP1 in patients with Alzheimer’s disease were negatively correlated with cognitive performance [11]. In addition to chromatin failure, oxidative mitochondrial injury can induce mtDNA oxidation, fragmentation, cytosolic redistribution, and acquisition of structural properties compatible with ZBP1-mediated inflammatory signaling. Defects in transcription termination and spliceosomal processing provide additional routes to persistent RNA duplexes and RNA:DNA hybrids [12].

Although arising from disparate causes, these disruptions can result in nucleic acids possessing shared structural attributes capable of engaging overlapping subsets of pattern-recognition receptors (PRRs) [13].

However, the molecular histories of these disruptions remain distinct. Heterochromatin failure exposes repeat-rich genomic regions; transcriptional read-through connects previously separated sequences; spliceosomal dysfunction preserves complementarity among intronic, exonic, and template-associated sequences; and mitochondrial injury modifies, fragments, and redistributes DNA that is normally confined within mitochondria [14]. We define the concept of “structural provenance” as describing this relationship, whereby the chemical and three-dimensional properties of an abnormal nucleic acid retain information regarding the type of quality-control failure that resulted in the generation or exposure of that nucleic acid [15].

The concept of structural provenance provides greater mechanistic detail than broad classifications such as double-stranded RNA, cytosolic DNA, or retrotransposon-derived RNAs. Substrates within these classes vary with respect to duplex continuity, chemical modification status, extent of protein coating, subcellular localization, conformational persistence, and receptor accessibility. Conversely, RNA–RNA duplexes, RNA:DNA hybrids, and damaged DNA may present convergent higher-order recognition surfaces despite their biochemical differences. Therefore, ZBP1 may selectively recognize a structurally privileged subset of endogenously produced nucleic acids rather than all nucleic acids classified solely according to their chemical characteristics [16].

For clarity, conformational proofreading and structural provenance, together with cooperative licensing, described below as the transition from Zα-domain recognition to productive RHIM-dependent complex assembly, are author-proposed interpretative frameworks for organizing experimentally supported observations into testable mechanistic sequences. These terms are not intended to represent established formal nomenclature or independently demonstrated biochemical processes.

### 1.2. Neural Context, Competitive Sensor Usage, and Hierarchical Evidence Requirements

Neural tissue may be particularly sensitive to disruptions in nucleic-acid homeostasis. Neurons undergo substantial transcriptional activity, extensive alternative splicing, activity-dependent chromatin remodeling, long-range RNA transport, and extensive cotranscriptional processing while remaining postmitotic, thereby reducing the dilution of accumulating molecular damage [17]. Microglia continuously survey damaged organelles, extracellular nucleic acids, protein aggregates, and other materials released from injured cells. Astrocytes orchestrate metabolic and interferon-responsive functions across neuronal and vascular compartments. Therefore, structurally similar substrates may produce different outcomes depending on the sensing cell they encounter and the surveillance mechanisms available within that cell [18].

Competitive occupancy also contributes to determining substrate fate. Endogenous double-stranded RNAs may be edited or bound by ADAR1, retained within the nucleus, degraded before productive ZBP1 engagement, or recognized by other PRRs such as MDA5 or PKR. Variations among ZBP1 proteoforms may also affect signaling competence, while the simultaneous regulation of nucleic-acid sensors and mechanisms limiting endogenous ligand accessibility by interferons adds another layer of complexity [19].

Biological outcome therefore depends on ligand structure and availability, receptor abundance, proteoform identity, subcellular compartmentalization, competitive sensor engagement, and signaling timing. Recognition must additionally be distinguished from execution. Association between a structurally compatible substrate and a Zα domain establishes receptor interaction; however, this interaction alone does not determine whether downstream signaling results in RIPK1-dependent inflammation, RIPK3–MLKL-dependent injury, apoptosis, or mixed inflammatory cell death [20].

Increased ZBP1 abundance, punctate staining patterns, detection of material with properties characteristic of Z-forms, or ZBP1 colocalization with death-associated proteins cannot independently establish involvement of a specific terminal pathway. Because much of the evidence supporting causal relationships has originated from preclinical models and experimental systems, translation to the human nervous system remains incomplete [21]. In particular, endogenous human brain-derived ZBP1 ligands, neural ZBP1 proteoforms, and neural cell-type-specific signaling complexes remain insufficiently characterized. These limitations should therefore be considered when extrapolating mechanistic findings from experimental models to human disease. Mechanistically relevant evidence must therefore demonstrate concordance among ligand structure, ZBP1 occupancy, signaling-complex composition and terminal effector activation within the respective cellular context [22].

Establishing only one of these elements demonstrates pathway association or competence rather than complete causal reconstruction. In conclusion, this review addresses: (i) nucleic-acid biogenesis and structural licensing; (ii) Zα-domain-dependent recognition by ZBP1; (iii) signaling-complex assembly via RHIM-dependent interactions; (iv) cell-type-specific responses within neural tissues; (v) competitive sensor utilization among ADAR1, MDA5, PKR, cGAS–STING-associated mechanisms, and ZBP1; (vi) limitations inherent to murine-to-human extrapolation; (vii) ZBP1 proteoform-dependent signaling competence; and (viii) criteria necessary for establishing causality. Particular attention is also directed toward distinguishing structural compatibility from identification as a bona fide ZBP1 ligand, ligand recognition from signaling commitment, pathway activation from causal dominance, and mechanistic plausibility from clinical therapeutic readiness.

### 1.3. Literature Search Strategy and Criteria for Study Selection

The current review was conducted as a mechanism-oriented narrative synthesis rather than a systematic review or meta-analysis. PubMed/MEDLINE, Scopus, Web of Science Core Collection, and Google Scholar were searched from database inception through August 2026, with iterative updates incorporated during manuscript preparation to capture newly published studies. Searches combined terms related to: (i) ZBP1/DAI; (ii) Z-DNA; (iii) Z-RNA; (iv) Z-nucleic acids; (v) RIPK1, RIPK3, and MLKL; (vi) RHIM-mediated signaling; (vii) ADAR1; (viii) MDA5 and PKR; (ix) cGAS–STING-mediated signaling; (x) interferon-mediated signaling; (xi) endogenous double-stranded RNA; (xii) RNA editing; (xiii) transcriptional dysregulation; (xiv) spliceosomal dysfunction; (xv) mitochondrial DNA release; (xvi) neuroinflammatory disorders; (xvii) Alzheimer’s disease; (xviii) tauopathy; (xix) traumatic brain injury; and (xx) neurodegenerative disorders. References cited within key primary studies and relevant reviews were also examined.

All studies identified through these searches were evaluated for relevance. Studies were considered particularly relevant when they reported mechanistic evidence concerning ligand biogenesis, chemical or structural characteristics, Zα-domain-dependent recognition, endogenous ZBP1 binding, RHIM-dependent complex assembly, genetic or pharmacological perturbation of relevant signaling pathways, downstream effector activation, or cell-type-specific responses. Human subjects, human-derived tissues, patient-derived samples, and human-relevant cellular systems were prioritized for translational interpretation when available. Correlative gene-expression and proteomic studies were included when they provided information regarding disease associations or cellular contexts, although these findings were interpreted conservatively.

Because of substantial heterogeneity among experimental systems, ligand-detection methods, signaling readouts and disease models, the evidence was synthesized qualitatively and no formal quantitative synthesis was attempted.

## 2. Molecular Biogenesis and Structural Licensing of Endogenous Z-Ligands

### 2.1. From Conformational Propensity to Ligand Competency

#### 2.1.1. Structural Thresholds, Avidity and Z-State Stability

A nucleic-acid molecule will be recognized as having structural competency for interaction with ZBP1 when it is able to achieve a suitable left-handed Z-form due to the combined influences of its sequence, duplex configuration, chemical modification, and environmental conditions. Sequence alone does not confer structural competency, nor does the ability to form a duplex. Alternatively, the fact that a duplex is located in the cytoplasm is insufficient to ensure ligand competency. Instead, successful interaction relies upon a duplex’s ability to enter and persist within a sufficiently stable Z-state for an adequate time and over a sufficient extent of its surface to enable effective interaction with the tandem Zα domains of ZBP1 [23].

Energy levels needed to effect the transition of a duplex from a double helix to a Z-form vary based on sequence and environmental factors. Selected combinations of repeating purine–pyrimidine tracts, particular CG-rich sequences, superhelical density, torsional strain induced during transcription, chemical modifications to cytidine residues, oxidative damage to guanine residues, electrostatic shielding of charge interactions, and protein binding all contribute to some degree to changes in the overall stability of the resulting structures [24]. Hence, sequence determines the tendency of a duplex to assume a Z-form; nevertheless, the establishment and continuation of this state are contingent upon the topology of the nucleic acid (superhelical density), chemical modification of the nucleic acid itself, localized molecular crowding, and the immediate protein environment [25].

Reductionism-based experimental design provides a first approximation of the structural thresholds required for effective interaction. Experimental models consisting of short duplexes exhibiting alternating CG sequences exhibit high affinity for the tandem Zα domains of ZBP1. As such, ligand competency is defined by avidity; i.e., the capacity of a nucleic acid to participate in multiple simultaneous interactions with either individual or groups of receptor molecules depends on the continued existence of a Z-state and the availability of the appropriate surfaces [26].

Thus, a transient Z-conformer may be capable of supporting single receptor-binding events while failing to support production of an oligomeric complex. Conversely, longer or topologically constrained nucleic acids may provide prolonged receptor proximity and alignment conducive to the generation of oligomeric complexes. The distinction between these states is critical because receptor binding and signaling competency represent distinct states [27].

#### 2.1.2. Transcript-Length-Scale Architectures of Endogenously Occurring Z-RNAs

RNAs found in cells are far more complex than those employed in reductionist experiments. Using genome-wide mapping methods after disrupting transcription-termination mechanisms, 1646 putative murine Z-RNAs were identified. Approximately 78% were produced from 3′ extension products beyond normal termination points; about 12% arose from cryptic intergenic transcription; and nearly 88% contained inverted endogenous retroelements capable of generating intramolecular RNA duplexes through pairing of LINE1 and B1/B2 SINE elements [28]. It is important to note that almost 87% of RNAs that exhibited antibody enrichment indicative of Z-form conformation were also associated with ZBP1, indicating a strong relationship between receptor occupancy and structural enrichment, although providing no direct evidence concerning ligand causality. A-to-I editing was detected in nearly 70% of the RNAs examined, indicating that many had developed duplex conformations and had thus encountered cellular RNA-surveillance pathways [29].

This represents a significant expansion relative to the previously accepted scale of investigation, from discrete sequence motifs displaying theoretically derived Z-form tendencies to architectural dimensions representing entire transcripts. An aberrantly elongated Nabp1 transcript measures approximately 45 kb—roughly sixteen-fold greater in length than its corresponding coding isoform—and contains two inverted LINE1 pairs associated with segments measuring approximately 1.8 and 8.8 kb [30]. Similarly, extended Gtpbp4 RNA contains inverted long terminal repeats flanking a region potentially forming predicted secondary structures. Bidirectional transcription initiated from cryptic intergenic sites produces Z-RNAs from the H2ac18/19 locus. In every case, the proposed Z-form segment resides within a substantially longer RNA, where total length, repeat orientation, folding rate, editing status, RNP composition, and nuclear-export characteristics determine whether complementary segments can form stable duplexes and remain available for interaction with their respective receptors [31].

#### 2.1.3. Validation Criteria Levelled by Strength of Evidence Supporting the Presence of Endogenous Ligands

Hence, competence as an endogenous ligand is independently demonstrated across at least four different scales. Primary sequence defines the intrinsic potential of a nucleic acid for assuming a Z-state; complementary architecture defines the maximal continuous length achievable between complementary strands; transcript topology defines how distant reverse-complementary elements can be placed within close spatial proximity; and, finally, protein occupancy defines what happens to the resultant structure (i.e., whether the structure is subjected to chemical modification, protected from degradation, remodeled, or otherwise processed) [32].

Therefore, computational Z-form propensity, conformationally selective enrichment, receptor occupancy, and signal-transduction capability should be regarded as independent yet progressively stronger validation criteria for the identification of ligands rather than as mutually interchangeable descriptors of ligand identity. Candidate ligands demonstrating agreement among molecular reconstruction data at the nucleotide level, chemical identity through nuclease digestion and/or enzymatic cleavage, structural competency utilizing orthogonal assay types, specific Zα-mediated ZBP1 occupancy employing biochemical assays, and substrate-specific signaling competency would constitute the highest-tier candidates for endogenous ligands. In the absence of convergence among these types of evidence, an anomalous nucleic acid would most appropriately be described as being compatible with a Z-form conformation or as a candidate ZBP1 ligand rather than as an unequivocally validated endogenous ZBP1 ligand [33].

### 2.2. Nuclear Boundary Failure Leads to Formation of Hidden RNA Topology

#### 2.2.1. Transcription Termination Failure and Capture of Repeats

When endogenous Z-RNAs are visible, it usually means that there have been disruptions in some or all of the nuclear mechanisms that regulate the size of transcripts, the sequences found in them, how they are processed, where they reside, etc. These include the regulation of heterochromatin structure (the condensed state), the cleavage/polyadenylation of mRNA, the splicing of pre-mRNAs, and how they are exported from the nucleus. The sum of these different regulatory mechanisms limits the amount of time for large stretches of complementary RNA segments to form and exist in the nucleus, in addition to limiting the existence of stable RNA:DNA hybrid molecules [34].

A good illustration of this concept is seen in the disruption of transcription termination. When factors that determine where RNA polymerase II stops transcription become disrupted, RNA polymerase II transcripts can continue past their usual 3′ ends and include downstream LINEs, SINEs, and long terminal repeats. This enables previously far-apart inverted repeats to be directly joined together in an RNA transcript and thus form paired structures extending over a distance greater than a kilobase [35]. Systems that fail to disrupt termination of host transcription do not produce nearly as much Z-RNA as those that disrupt termination. However, certain viral proteins that inhibit termination, as well as pharmacological treatments affecting the ability of cleavage and polyadenylation specificity factors to interact with their target mRNAs, lead to similar ligand-producing conditions [5]. Therefore, cleavage and polyadenylation serve not only as mechanisms for RNA processing but also as structural barriers preventing incorporation of distantly located repetitive elements into mature host transcripts able to maintain long-lasting complementary architectures [36].

#### 2.2.2. Relaxation of Heterochromatin and Release of Repetitive Elements

Relaxation of heterochromatin represents a second pathway leading to the formation of latent Z-form-compatible topologies by increasing transcriptional access to genomic regions rich in repetitive DNA sequences. Pathological tau expression has been shown to correlate with disturbances in H3K9me3-enriched chromatin, decreased heterochromatin organization due to loss of HP1 association, elevated transcription of mobile genetic elements, and accumulation of Z-RNA in tau-expressing neurons [37]. However, the key mechanistic unit is not necessarily the individual repeat family itself but rather the entire transcript in which the repeat resides. Thus, autonomous retroelement transcripts, host–repeat chimeras, bidirectionally transcribed RNAs, and RNAs spanning multiple kilobases containing inverted repeats can all accumulate after disruption of heterochromatic barriers. However, the degree to which these RNA species contain continuous duplexes or compatible conformations will vary greatly. The relationship between sequence connectivity and repeat orientation within the full-length RNA molecule could therefore offer more mechanistic insight than simply measuring repeat content [38].

#### 2.2.3. Aberrant Pre-mRNA Processing and Retained Introns/Unresolved Complementary Regions

Aberrant pre-mRNA processing represents a third pathway through which latent complementary topology can be preserved. Exonic and intronic RNAs formed through aberrant splicing can possess the capability to form Z-compatible duplex structures within the nucleus. Upon disruption of pre-mRNA processing and subsequent export of mis-spliced RNAs into the cytosol, retained introns and unprocessed complementary regions become exposed to cytosolic sensors such as ZBP1 [39].

Furthermore, aberrant splicing can create structurally unique nucleic-acid structures. For instance, a Zα-derived probe has identified RNA:DNA hybrids present in the nucleus. Additionally, structural modeling demonstrates that short hybrid duplexes can assume a left-handed geometry consistent with binding by a Zα domain [40]. It is essential to note that these substrates represent distinct classes of nucleic acids. Hybridized RNA:DNA strands are more likely to persist near sites of active transcription or at intermediate states of cotranscriptional resolution, while RNA–RNA duplexes are more likely to migrate to the cytosol following impaired processing. Consequently, maintenance of proper spliceosome assembly may restrict conformational danger via several related mechanisms, including resolution of nascent transcript–template associations, elimination of intronic complementarity, correct RNP assembly, and limitation of immature RNA transport [41].

Therefore, termination failure, heterochromatin collapse, and aberrant splicing can be viewed as three separate types of nuclear boundary failures. Each type represents a method by which coding transcripts can become associated with additional repetitive elements; heterochromatin collapse increases transcriptional accessibility to previously cryptic loci; and aberrant splicing maintains nascent intronic, exonic, and template-associated complementarity. Regardless of the specific failure event that occurs, each preserves a structural record reflecting the nuclear process that was compromised. These records can be measured based upon the chemical properties of the duplex, architectural features of the transcript, and intracellular trafficking characteristics [42].

### 2.3. Oxidative and Spatial Licensing of Mitochondrial DNA

#### 2.3.1. Oxidative Modification, Fragmentation and Nucleoid Escape

The way in which mitochondrial DNA (mtDNA) achieves competency to interact with ZBP1 is very different from that of nuclear RNA. mtDNA is contained within mitochondria as part of nucleoid assemblies composed of DNA associated with protein molecules; it has a circular topology and is isolated from nearly all cytoplasmic nucleic-acid receptors by virtue of being located within mitochondria. Therefore, mtDNA origin alone does not provide competency to interact with ZBP1. For mtDNA to become a suitable substrate for interaction with ZBP1, there must be disruption of nucleoid organization, chemical modification of the DNA, fragmentation, movement out of the mitochondria and into the cytoplasm, and persistence of a higher-order DNA structure in the cytoplasm that is accessible to the receptor [43].

When microglial cells are exposed to amyloidogenic stress conditions, elevated levels of ROS generated within mitochondria lead to oxidation of guanines in mtDNA, fragmentation of mtDNA, and eventual release of mtDNA into the cytosol. The released mtDNA contains regions with Z-DNA-associated properties and binds to ZBP1, indicating that a structurally compatible substrate for this receptor exists. However, this study cannot confirm whether each mtDNA molecule released from the mitochondria maintained the same conformation as that present at the time of release [44].

Therefore, the potential candidate ligand is a portion of mtDNA that has been chemically altered and spatially relocated in a manner that increases its physical accessibility to cytoplasmic receptors. Oxidative modifications could influence the ability of mtDNA to serve as a substrate for interaction with ZBP1 through several mechanisms. The formation of 8-oxoG creates changes in base-stacking interactions and alterations in local helical stability due to oxidative modification. Additionally, oxidative strand breaks cause fragmentation. Fragmentation reduces the topological constraints created by the circular nature of mtDNA and allows greater physical accessibility to cytoplasmic sensors [45]. Disruption of nucleoids and escape of DNA from proteins that would normally limit access to receptors allow a shift toward states compatible with receptor interaction. It should be noted that these processes do not eliminate other possible structural arrangements that could result in equivalent biochemical behaviors. Thus, conformational changes directly observed in experiments should be differentiated from structures assigned based solely upon selective binding, enrichment, or immunoreactivity [46].

Therefore, four separate checkpoints can be recognized in the production of mitochondrial ligands: oxidative modification, fragmentation, exit of mtDNA from the mitochondria into the cytosol, and stabilization of at least one higher-order structure compatible with receptor interaction. It is possible that these events do not have to proceed in a strictly sequential fashion. Stress experienced by mitochondria could produce mtDNA release without the creation of a substrate compatible with a Z-form, and oxidative modification could create chemically altered DNA without generating fragments capable of exiting the mitochondria. In such cases, mtDNA associated with ZBP1 should be designated as Z-DNA only when structural data confirm this designation [47].

#### 2.3.2. Physical Access to Receptors and Competition Among Pathways That Sense DNA

This differentiation is important because cytoplasmic mtDNA can activate multiple innate immune responses, including cGAS, TLR9, and NOD-like receptor inflammasome-associated pathways. Determinants influencing selection among these pathways include, but are not limited to, fragment length, degree and type of oxidation, strandedness, associations with proteins, location within the cell, persistence, and higher-order conformation. Therefore, determining which pathway or pathways are activated is unlikely to depend solely on the source of mtDNA, i.e., its mitochondrial origin [48]. Transfer via extracellular vesicles represents another level of spatial separation. Extracellular vesicles produced by neurons can contain mtDNA that is delivered to microglia, thus allowing the ligand-producing neuron to be separated from the sensing microglia. However, delivery of mtDNA from neurons to microglia does not prove that the mtDNA existed in a Z-form configuration during transport, nor does it prove that each mtDNA molecule delivered is competent to interact with ZBP1 [49].

Therefore, determination of ligand identity must be reconstructed across both molecular and intercellular scales. Thus, nuclear and mitochondrial substrates acquire competency to act as ligands for ZBP1 through distinct yet convergent pathways. Nuclear substrates arise when barriers to transcription and RNA processing fail to preclude maintenance of complementary sequence pairs within RNA–RNA duplexes or RNA:DNA hybrids [50]. Mitochondrial substrates arise when oxidative modification, fragmentation, nucleoid disruption, and membrane escape transform a previously protected chromosome into an accessible substrate in the cytosol [51]. Table 1 is intended to synthesize these ligand-defining variables across molecular contexts and to distinguish mere nucleic-acid accumulation from the acquisition of receptor-competent architecture. In all cases, ligand abundance is insufficient; recognition instead arises from the convergence of defined sequence architecture, chemical history, spatial trajectory, and persistent nucleic-acid conformation.

Structural licensing creates a receptor-accessible ligand, but does not explain how recognition becomes signalling. Section 3 therefore examines how Zα engagement reorganises ZBP1 and initiates RHIM-dependent signal-complex assembly [61].

## 3. From Zα Recognition to RHIM-Encoded Signalling

### 3.1. Cooperative Zα Recognition Provides an Assembly Threshold for Activation

#### 3.1.1. Ligand-Dependent Receptor Association and the Assembly Threshold

The information contained in nucleic-acid conformation and structure is transmitted to ZBP1-dependent signalling through two structurally distinct regions of ZBP1. These include the N-terminal Zα domains, which recognize nucleic acids with left-handed geometry and the RHIMs, which facilitate protein–protein interactions involving RHIM-containing proteins. Thus, Zα-occupied receptors represent recognition events but do not themselves activate the receptor. For a receptor to be activated, it must remain bound to a nucleic acid for a sufficient duration, achieve a sufficiently high local density, and adopt compatible orientations relative to other receptors to allow cooperative protein assembly [62].

Research employing biophysical techniques has provided evidence supporting a threshold model of complex formation via cooperative assembly. Isolated tandem Zα domains dimerized in a concentration-dependent fashion and preferentially interacted with a DNA duplex composed of alternating C–G residues approximately ten base pairs in length. This indicated that Z-DNA and Z-RNA could serve as multivalent organizing surfaces rather than as single receptor-binding epitopes [63]. Therefore, simply being able to bind to a receptor based on affinity is not sufficient to create competent conditions for initiating signalling. Instead, factors such as duplex length, whether the nucleic acid maintains its conformation throughout its length, the number of potential binding sites available on each ligand surface, local receptor density, and the duration for which receptor–ligand association is maintained all determine whether an initial recognition event proceeds toward productive assembly [64].

#### 3.1.2. Functional Asymmetry Between Zα1 and Zα2

Significant functional differences exist between the two tandem Zα domains. Mutations in Zα2 completely ablated RIPK1 recruitment and significantly impaired the formation of ZBP1–RIPK3 complexes during human necroptosis stimulated by nucleic acids. Additionally, mutations produced similar impairments in RIPK1 recruitment and the formation of ZBP1–RIPK3 complexes in murine cells generated with domain mutants. However, the impairment was less severe than that observed when utilizing murine ZBP1 within human cell lines or murine cellular systems generated with domain mutants [65]. The results thus provide substantial support for a significant role of Zα2 and the proximally located signalling RHIM in triggering ligand-induced assemblies, whereas the contributions made by Zα1 and the distally located RHIM to productive assembly appear to be relatively smaller. Consequently, the findings support a directional recognition-to-assembly pathway whereby Zα2 supplies a large portion of the structural information relating to the nucleic acid and the proximal RHIM converts this information into adaptor recruitment [66].

That being said, studies using inducible human ZBP1-overexpression systems demonstrated both delayed cell death and diminished expression of inflammatory genes when either Zα domain was mutated. However, when elevated levels of mutant ZBP1 were maintained for extended periods, delays in cell death and decreased expression of inflammatory genes were greatly diminished [67]. It follows that whether a given system reaches an assembly threshold is directly related to the amount of receptor present at the time the system is activated. If the level of receptor present is at or near physiological levels, activation of productive signalling depends strongly on conformational recognition [68]. On the other hand, if receptor abundance is markedly increased as a result of forced overexpression, mass action may promote assembly of the system even though individual receptors have a diminished capability to recognize their respective ligands. Consequently, phenotypes observed under conditions of overexpression should be considered indicative of the inherent propensity of the system to produce assembly products rather than as direct representations of productive receptor activation in vivo [69].

#### 3.1.3. Cooperative Licensing: An Example of the Recognition-to-Assembly Process

In this review, we refer to this process as cooperative licensing. Simply stated, a ligand that adopts a left-handed conformation does not automatically initiate a productive signal through the binding of a single receptor molecule. Instead, recognition increases local receptor density, residence time, and spatial compatibility among multiple molecules of ZBP1 until weak intermolecular interactions become collectively favorable. Herein lies our definition of cooperative licensing. In this context, nucleic-acid conformation is converted into local receptor organization, and RHIM networks stabilize only once an assembly threshold has been attained [70].

### 3.2. RHIM Nucleation Converts Receptor Association into a Signal Transduction Commitment

#### 3.2.1. Ligand Binding to Receptors Creates RHIM-Dependent Supramolecular Assembly

Receptor association with the ligand and the subsequent creation of an activated signalosome involve alterations in supramolecular organization. Ligand-enriched condensate states have been demonstrated to occur in vitro using purified Zα-containing peptides. However, the physical characteristics of the condensates were shown to vary depending upon the experimental conditions. For example, synthetic Z-DNA created condensates that exhibited limited fluorescence recovery and behaved as gels, while condensates generated from Zα regions during viral infection were found to have greater internal exchange [71]. Fluorescence recovery of full-length ZBP1 puncta was lower, indicating that regions external to the Zα domain contributed to stabilization of the assembly. Importantly, receptor enrichment, gel formation, intracellular puncta, and cross-β polymeric structures are not obligatory stages of a single liquid-to-solid phase transition. Rather, each represents an experimentally distinct form of higher-order organization [5].

The primary structural mechanisms through which receptor enrichment can convert into signaling commitment are provided by the RHIMs. Conserved hydrophobic core regions exist within relatively disordered regions of RHIMs and can participate in β-sheet-rich heteromeric assemblies with other RHIM-containing proteins, including RIPK1 and RIPK3. The IQIG tetrad (aa 206–209) is important for both RIPK-family protein association and the formation of amyloid-like fibrillar structures that facilitate downstream signaling from the proximal human ZBP1 RHIM [72]. Fibrillar material exhibiting diffraction peaks at approximately 4.7 Å and 9.4 Å has been detected using isolated ZBP1 RHIM fragments. Additionally, these fragments promoted RIPK1 RHIM polymerization, as evidenced by thioflavin T assays. Collectively, these studies suggest a templating mechanism in which a ZBP1-associated RHIM recruits and organizes compatible adaptor RHIMs [73].

A key feature of the polymerization process is that it creates properties that cannot be achieved through isolated pairwise interactions. Avidity increases through multivalent contacts and dissociation decreases; a few ligand-bound receptors can recruit multiple adaptor molecules; and a preformed RHIM nucleus can serve as a directional template for the incorporation of additional RHIM-containing adaptors [74].

#### 3.2.2. Assembly of Higher-Order Structures and Indications of Downstream Signaling Commitment

In theory, polymerization could create hysteresis, such that once an adaptor network has formed and developed sufficient kinetic stability, signaling may continue even if the level of initiating ligand fluctuates. While this concept remains speculative with regard to ZBP1, it offers a possible explanation for the nonlinear relationship between receptor density and biological response observed in many experimental systems [75].

The geometry-to-assembly transition comprises at least four experimentally distinguishable stages: Zα-dependent receptor aggregation, formation of a RHIM-competent nucleus, incorporation of RIPK1 and/or RIPK3, and activation of the appropriate scaffolding or kinase functions. Formation of a punctum alone cannot confirm progression to the final stage of this process. Similarly, detergent insolubility or thioflavin reactivity indicates higher-order structure but does not identify the adaptor(s) recruited or the final effector(s) utilized [76].

Therefore, receptor aggregation mediated by Zα, assembly mediated by RHIMs, recruitment of adaptor(s), and activation of terminal effector(s) represent progressively stronger levels of evidence for signaling commitment rather than equivalent indicators of pathway activation. Thus, confident identification of a signaling complex requires demonstration of its molecular composition coupled with evidence of the relevant downstream catalytic or effector activities.

#### 3.2.3. Spatial Distribution and Species-Specific Regulation of Distal RHIM Regions

**Figure 1:**
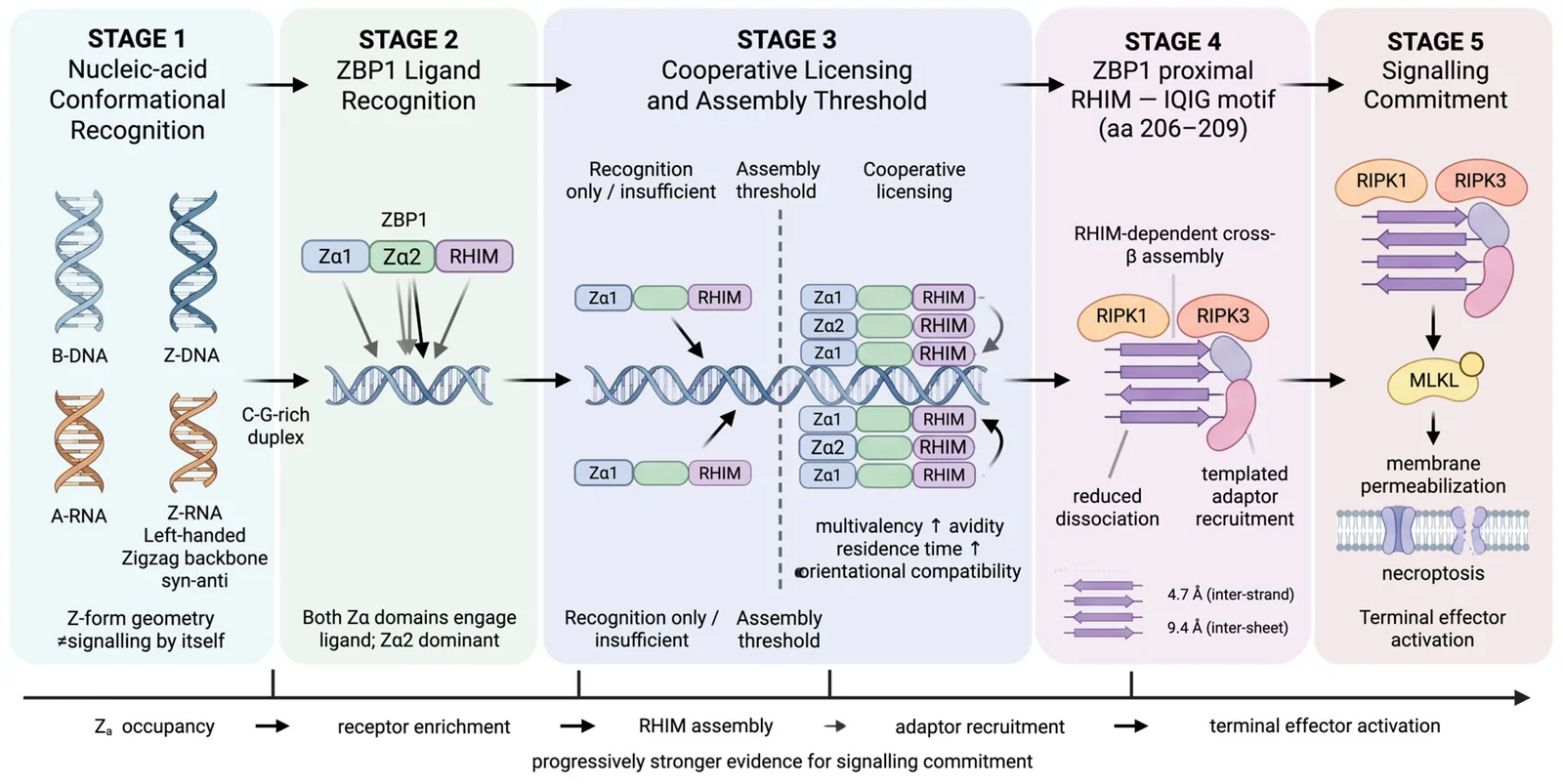
Cooperative Zα recognition establishes an assembly threshold for ZBP1 signalling. Canonical B-DNA/A-RNA and left-handed Z-DNA/Z-RNA are contrasted to illustrate structural ligand recognition by the Zα domains of ZBP1. Ligand engagement increases receptor density, residence time, and orientational compatibility, enabling cooperative licensing once a critical assembly threshold is reached. The proximal RHIM, including the IQIG motif (aa 206–209), then promotes higher-order cross-β assembly and recruitment of RIPK1/RIPK3. Productive signalling can subsequently activate MLKL and drive necroptosis. The lower axis summarizes progressively stronger evidence for signalling commitment: Zα occupancy, receptor enrichment, RHIM assembly, adaptor recruitment, and terminal effector activation.

Distal regions of human ZBP1 modulate the conversion to productive assembly in a species-specific manner. Three RHIM-containing regions within human ZBP1 were recently demonstrated to contribute significantly to cytoplasmic assembly and cell death. The first RHIM was demonstrated to interact with RIPK1 and act as the primary site of interaction, whereas the two more C-terminal RHIM2/RHIM3 regions enhanced productive engagement among RHIM-containing domains [67]. Transfer of the distal region of human ZBP1 into murine ZBP1 resulted in transfer of the increased activity characteristic of human ZBP1 to the murine receptor. In contrast, truncation of the C-terminal region of murine ZBP1 resulted in constitutive activation, indicating that the corresponding region within murine ZBP1 can act as a regulatory region. Therefore, the distal region of ZBP1 does not universally activate or inhibit signaling. Rather, its effect depends upon intramolecular associations between regions, the spatial arrangement of RHIMs relative to one another, receptor architecture, and compatibility with adaptor proteins within a particular species [73]. The stepwise transition from nucleic-acid binding to signalosome formation is represented as a cooperative assembly model for ZBP1 activation in Figure 1.

### 3.3. Species-Specific RHIM Grammar Defines Which Adaptors Are Recruited

#### 3.3.1. Comparison of RIPK1-Dependent Necroptotic Signaling in Murine and Human Cells

Necroptotic signaling initiated by ligands requires RIPK1-dependent detergent-insoluble polymer formation and RIPK3 phosphorylation in human cells. As a result, acute degradation of RIPK1 or inhibition of RIPK1 kinase activity blocks necroptotic signaling in multiple human cell types. On the other hand, there are marked differences in RIPK1 requirement for murine cells undergoing necroptosis. This is illustrated by the ability of murine ZBP1 to associate with RIPK3 and initiate necroptosis independently of RIPK1, whereas human ZBP1 requires RIPK1 for this association. However, murine RIPK1 was shown to colocalize with murine ZBP1, RIPK3, and the remaining components of the necrosome [77].

Although ZBP1 proteins from either species retained many characteristics of their native hosts when expressed in cells of the other species, a species-specific difference related to RIPK1 dependency was noted. Specifically, murine ZBP1 expressed in human cells showed the human-like RIPK1 dependency characteristic of the human cellular environment. In contrast, human ZBP1 expressed in murine cells did not retain the RIPK1-dependent necroptotic response typical of its human counterpart. Together, these results indicate that one major determinant of species-specific differences in RIPK1 dependency relates to the RHIM-domain sequence of RIPK3 [78,79]. Substitution of the human RHIM domain of RIPK3 with the corresponding murine RHIM domain resulted in reduced RIPK1 dependency during complex formation. A possible molecular basis for these differences has been modeled structurally. The model suggested that the position of the murine F442 residue within the hydrophobic pocket of ZBP1 was more favorable than that of the corresponding human I452 residue. Additionally, three predicted hydrogen-bonding interactions between murine RIPK3 and ZBP1 were absent from the modeled human complex [80]. Since structural models require experimental validation, the demonstrated functional effects provide evidence that even relatively minor changes in RHIM-domain sequences can substantially alter the organization of signaling complexes. Therefore, it is possible that RIPK1 acts as a bridging protein to compensate for decreased ZBP1–RIPK3 compatibility in human cells [81].

#### 3.3.2. Context-Dependent Adaptor Routing Determines Cell-Fate Decisions

Using inducible-expression systems, distinct mechanisms of activating human ZBP1 have also been studied. In these systems, human ZBP1 induced RIPK1-dependent cell death with reduced RIPK3 participation. In addition, RIPK1 served as a scaffold to support FADD–caspase-8-associated apoptosis, whereas RIPK1 kinase activity supported necroptotic execution. All three RHIM-containing regions of human ZBP1 were necessary for efficient complex assembly under these conditions [82]. Thus, while these findings do not rule out the previously reported RIPK1-bridged ZBP1–RIPK3 mechanism utilized during ligand-induced viral necroptosis, they suggest that varying levels of receptor abundance and/or activation modes can modulate adaptor stoichiometry and the order of pathways activated [65].

Studies using genetic approaches in murine systems have also shown that ZBP1 can specify multiple cell fates. Overexpression of constitutively active forms of ZBP1 can lead to both RIPK3–MLKL-mediated necroptosis and RIPK1–FADD–caspase-8-mediated apoptosis. The latter apoptotic pathway is dependent on the RHIM domain of RIPK1 but does not require RIPK1 kinase activity [83,84]. Elimination of the inflammatory phenotype required disruption of both apoptotic and necroptotic pathways. Therefore, based on the above information, RIPK1 should be considered a multifunctional signaling molecule that can perform at least three different functions: (1) serve as a RHIM-containing assembly component, (2) act as a scaffold for recruitment of apoptotic machinery, and (3) in certain contexts, act as an active kinase [85].

Taken together, these data strongly support a species-specific and context-dependent RHIM grammar. Ligand interaction through Zα determines whether ZBP1 becomes competent for assembly; the spatial relationship between the ZBP1 RHIM domains determines which adaptor interfaces become available; the sequences and compatibility of adaptor RHIM domains determine which higher-order assemblies can form; and the scaffolding and catalytic activities of RIPK1, RIPK3, caspase-8, and MLKL determine which pathway or pathways will ultimately be executed. Therefore, neither receptor or adaptor expression or localization, punctum formation, RIPK recruitment, nor detection of any single signaling component can definitively identify necroptotic commitment [5].

Thus, ZBP1 is best viewed as a conformationally licensed platform for higher-order assembly rather than as a receptor obligatorily linked to a single terminal pathway. Apparently similar ZBP1-containing complexes can yield different biological outcomes depending upon species, cell type or state, adaptor availability, receptor density, and the presence or absence of signaling machinery within the responding neural cell [86].

## 4. Encoding of Neuronal States and Intercellular Transmission of ZBP1 Signals

### 4.1. Constraints of Neuronal Execution by Cellular Structure and Receptor Dosage

#### 4.1.1. Neuron-Intrinsic Execution and Structural Vulnerability

Cellular structure and receptor dosage are central components in assessing how neural cell states encode intercellular transmission of ZBP1 signals, since neurons have limited regenerative capabilities. They primarily exist in a postmitotic state that limits the replacement of lost neurons in existing neural circuits. To maintain their function, neurons rely on maintaining membrane integrity, synaptic organization, axonal transport, and the distribution of mitochondria throughout highly polarized structures. As such, ZBP1 signalling in neurons should be evaluated beyond the confines of somatic cell death to include early structural changes (e.g., synaptic depletion and neurite degeneration), which affect long-range connectivity [86].

Tauopathy provides strong preclinical evidence for ZBP1 execution due to neuron-intrinsic activity. Tau-expressing neurons contained endogenously produced Z-RNA that triggered a ZBP1-dependent RIPK3–MLKL pathway. Disruption of either ZBP1 recognition or downstream RIPK3 signalling was shown to reduce neuronal death in experimental models. Zbp1 haploinsufficiency in PS19 mice resulted in fewer pMLKL-positive neurons, less neuronal and glial pathology, and better cognitive performance [60,87]. These protective effects persisted for up to two years of age, indicating that ZBP1 contributes to pathology that develops during the later stages of disease progression and not only during initial disease onset. Higher levels of ZBP1 in excitatory neurons from individuals with Alzheimer’s disease correlated inversely with cognitive ability, further demonstrating relevance to human disease but not providing direct evidence for human neuronal necroptosis [88].

#### 4.1.2. Receptor Dosage and Threshold-Dependent Neuronal Damage

Another mechanism associated with reducing receptor dosage is that productive signalling occurs via cooperative receptor assembly. It would thus be difficult to argue that there is a linear relationship between ZBP1 concentration and neuronal damage. Cooperative interactions among receptors are required for effective signalling. The likelihood that ligand-bound receptor complexes reach the threshold required for recruiting effectors is decreased when the number of receptors present is diminished. One potential rationale for why haploinsufficient mice exhibit a lower incidence of damage is therefore based on this model; however, receptor stoichiometry has yet to be demonstrated experimentally at the level of individual neurons [89].

Another mechanism associated with reducing receptor dosage is that productive signalling occurs via cooperative receptor assembly. It would thus be difficult to argue that there is a linear relationship between ZBP1 concentration and neuronal damage. Cooperative interactions among receptors are required for effective signalling. The likelihood that ligand-bound receptor complexes reach the threshold required for recruiting effectors is decreased when the number of receptors present is diminished. One potential rationale for why haploinsufficient mice exhibit a lower incidence of damage is therefore based on this model; however, receptor stoichiometry has yet to be demonstrated experimentally at the level of individual neurons [89]. Where within the subcellular domain of the neuron ZBP1 exerts its lethal effects remains unknown. Detection of pMLKL demonstrates a certain degree of pathway activation; however, it does not indicate whether plasma-membrane damage is localized to specific regions of the neuron, i.e., the soma, dendrites, axons, or synapses. Local MLKL oligomerization could potentially disrupt ion permeability, calcium homeostasis, or organelle trafficking prior to overt plasma-membrane rupture [90]. Since neurons are extremely polarized, localized injury to an individual compartment could lead to withdrawal of synapses or proximal degeneration before somatic death. There is currently no empirical evidence available to support this model, and therefore it should remain a testable hypothesis until localization data become available for endogenous ZBP1 complexes, active RIPK3 and oligomeric membrane-associated MLKL [91].

#### 4.1.3. Autonomous Execution by Individual Neurons Versus Secondary Glial Injury

It will also be essential to differentiate between cases in which ZBP1 signalling is executed within a single neuron and those in which secondary injury results from inflammation in adjacent glial cells. A neuron may execute ZBP1 signalling independently, subsequently release damaged nucleic acids detected by microglia, or experience secondary injury resulting from inflammation generated in surrounding cells. These processes may occur concurrently; however, they require separate empirical verification. Modification of the receptor in isolated neurons tests autonomous execution; modification of the receptor in microglia tests transcellular sensing; and modification of nucleic-acid release tests the process of intercellular communication. Empirical demonstration that a given intervention protects at the tissue level does not allow us to determine which cellular compartment contained the relevant receptor activity [92,93].

### 4.2. Microglial ZBP1 Spans Both Adaptation to Inflammation and Destructive Commitment to Cell Death

#### 4.2.1. Non-Lethal and Adaptive Inflammatory States

Microglial ZBP1 can elicit multiple biological effects when stimulated, including alterations in gene expression, phagocytic activity, and cellular viability. Available neural models describe at least three general states of microglial ZBP1 signalling: (1) chronic, non-lethal inflammation; (2) transiently active protection against harmful stimuli; and (3) lethal, pro-inflammatory cell death. These states can occur under varying experimental conditions, and thus it would be inaccurate to consider them simply as alternative representations of the same underlying biological mechanism [94].

Therefore, ZBP1-mediated inflammatory transcription, adaptive microglial activation, and lethal cell-death commitment are better viewed as mechanistically distinguishable biological outputs, rather than increasingly strong expressions of a single biological pathway. The primary mechanisms responsible for ZBP1-mediated microglial recognition of Z-forms of mitochondrial DNA and subsequent RIPK1-dependent inflammatory signalling were found to contribute to the development of amyloid plaques. Deleting Zbp1 in mice and inhibiting RIPK1 pharmacologically resulted in significant reductions in amyloid-plaque-associated neuroinflammation, amyloid burden and behavioral dysfunction [60]. An important finding from this study was that the most well-characterized function of the ZBP1-mediated signalling cascade was regulation of inflammatory transcription, rather than mediation of necrosis through RIPK3–MLKL. As such, in this model, ZBP1 appeared to modulate the local inflammatory environment surrounding the amyloid plaque while still allowing the ZBP1-containing microglia to remain alive and continue to perform their functions [84].

#### 4.2.2. Transitional Activation from Adaptive to Destructive Signalling

A model demonstrating temporal progression between these programmed responses is mild traumatic brain injury (mTBI). The release of mtDNA from injured neurons into extracellular vesicles permitted mtDNA to interact with microglial ZBP1. This interaction initiated a TBK1–IRF3 pathway, resulting in the production of IL-6 and TNF. Mice lacking Zbp1 had decreased acute cortical microglial activation after mTBI when compared with wild-type mice [95]; however, the absence of ZBP1 did not decrease astrocyte activation after injury. Additionally, mice lacking ZBP1 developed greater impairments in hippocampus-dependent memory functions following mTBI. Collectively, these results suggest that initial ZBP1-mediated microgliosis contributes to damage restriction, debris removal, and recovery processes and is not necessarily detrimental per se [90].

On the other hand, severe TBI can result in destruction of the activated microglial cell. Synergistic actions of heme and TNF-α in the initiation of a ZBP1-mediated mixed programme consisting of inflammasome activation, apoptosis, and necroptosis, termed PANoptosis, have been reported. The deubiquitinase USP4 interacts with and deubiquitinates ZBP1, thereby stabilizing the receptor [96]. AKT phosphorylates USP4 and maintains USP4 protein stability. Vialinin A-treated cells showed reduced USP4 levels, increased ZBP1 turnover, and decreased microglial death. Vialinin A treatment also improved functional outcomes in models of traumatic injury. Finally, higher levels of USP4 mRNA correlated with poorer patient outcomes in cases of severe traumatic brain injury [97].

Collectively, these data support the notion that the fate of microglial cells depends upon receptor proteostasis, including the balance between receptor stabilization and degradation, as well as the duration during which signalling-competent ZBP1 persists relative to the capacity of microglial cells to eliminate the initiating ligand and limit downstream effectors [98].

#### 4.2.3. ZBP1 Proteostasis and Therapeutic Timing

These continua may provide important considerations when interpreting therapeutic interventions. When microglial cells exist in a protective or adaptive surveillance state due to transient receptor activity, interference with ZBP1 could suppress a beneficial response. Alternatively, if therapeutic intervention occurs once receptor stabilization and prolonged downstream signalling have developed, termination of a pathological inflammatory programme and preservation of the microglial population could potentially be achieved [99]. Therefore, therapeutic timing may be as important as the choice of target(s). To determine at what point therapeutic intervention becomes beneficial, specific knowledge regarding the state-dependent molecular checkpoints within this continuum will be required, including: (1) ZBP1 ubiquitination status; (2) RIPK1 kinase activity; (3) RIPK3–MLKL complex formation; (4) caspase activation; and (5) inflammasome formation.

Table 2 aims to summarize the molecular states of microglial ZBP1 signaling by defining the sequential checkpoints that lead to one of three final outcomes of receptor activation: supportive or adaptive inflammation, sustained inflammatory signaling or irreversibly committed cell death.

### 4.3. The Physical Separation Among Producers, Sensors, and Effector Cells Represents a Producer–Sensor–Effector Circuit

#### 4.3.1. Transferring Ligands Using Extracellular Vesicles

The ZBP1-dependent signalling cascade in response to mtDNA released via EVs from neurons to microglia does not have to occur entirely within one cell. Therefore, EVs allow mtDNA produced or released by neurons to be transferred to microglia, which then respond to their local environment through either inflammatory or reparative pathways. As a result, the biologically relevant functional unit may best be described as a producer–sensor–effector circuit instead of simply describing the mechanism as a cell-autonomous pathway [44,95].

In addition to providing a means to transfer mtDNA between cells, EV-mediated transfer may physically modify the mtDNA-based ligand during packaging within EVs, during transport from neuron to microglia, after internalization into the microglial cell, and throughout intracellular processing. The modifications made to the ligand during this process may include changes in fragment size, degree of oxidation, associated proteins, and ligand bioavailability within various cellular compartments [107]. Therefore, when mtDNA-derived signals originating from neuronal mitochondria arrive at ZBP1 in a microglial cell, the mtDNA-based ligand may have been structurally altered from its original state. While the ligand maintains sufficient structural competency to bind to ZBP1, there is no evidence confirming the precise conformation of the ligand at the time it first engaged ZBP1 [108].

#### 4.3.2. Factors That Influence the Outputs of the Circuits

The biological outcomes of these circuits will depend on the duration for which the circuit operates and the spatial relationship between the producers and sensors within the circuit. If microglia rapidly sense damage caused by the accumulation of amyloid-β or dysfunctional mitochondria and can direct repair, this rapid detection may help localize damage and guide reparative responses. However, if ligands are continuously delivered into the circuit over prolonged periods, these persistent signals may sustain expression of genes involved in inflammation in microglia, maintain receptor stability, or even cause microglial cell death. Microglia surrounding plaques continuously detect amyloid-induced stress and mitochondrial damage for extended periods [109]. Similarly, long-projecting neurons can propagate organelle dysfunction along their extensive axonal projections to anatomically distant regions. Therefore, localized pathology may be influenced by several factors, including where the ligands are produced, the distribution of receptor-competent cells, and the efficiency of removing intercellular signals [110].

Therefore, the output resulting from neural ZBP1 receptors can be described as a function of at least four different factors, including the type of cell that senses the ligands, the timing of activation relative to other pathological events occurring in the tissue, the rate of ZBP1 degradation and receptor proteostasis, and the spatial relationship between where the ligands are produced and where receptor recognition occurs [111]. Signalling in neurons can convert molecular stress into permanent structural loss; signalling in microglia can activate adaptive responses, generate chronic inflammation, or induce death of activated microglia; and signalling between cells can convey information about the initiating pathological stimulus to anatomical regions distant from those originally affected [112].

#### 4.3.3. Establishing Initiating, Amplifying, and Terminal Execution Roles

Using a producer–sensor–effector framework also provides an approach to experimentally define whether ZBP1 serves primarily in an initiating role, an amplifying role, or a terminal execution role in circuits contributing to neural diseases. Demonstrating that transient disruption of ZBP1 before downstream pathological changes such as inflammation or degeneration prevents subsequent development of the pathway would suggest that ZBP1 serves primarily in an initiating role [113]. Demonstrating that delayed disruption of ZBP1 diminishes progression of pathology while the initiating pathological stimulus continues to exist would suggest that ZBP1 serves primarily in an amplifying role. Demonstrating that ZBP1-dependent signalling directly activates downstream effectors leading to structural damage in responding cells would suggest that ZBP1 serves in a terminal execution role [73].

These hypotheses can be tested using cell-type-specific genetic modification techniques, temporal control of receptor depletion or inhibition, blockade of ligand transfer, and analysis of the spatial relationships between endogenous ZBP1 complexes and RIPK3, MLKL, caspases, or inflammatory outputs in situ. Protection at the tissue level cannot definitively establish ZBP1’s causal position within a circuit [114].

ZBP1 signalling within neural cells is therefore determined not only by ligand specificity and receptor density but by the convergence of multiple variables, including cell type, receptor dosage, subcellular localization, receptor proteostasis, duration of activation, and ligand transfer between cells. These variables influence whether ZBP1 predominantly acts as an adaptive sensor, sustained inflammatory amplifier, or irreversible executioner [5].

Cellular context, however, does not predetermine which sensor will recognize aberrantly modified nucleic acids. Multiple competing proteins may edit, compartmentalize, or recognize the same substrate before assembly by ZBP1. Section 5 will describe how ADAR1, MDA5, PKR, and related mechanisms partition endogenously available nucleic acids to establish an ordered hierarchy of innate immune surveillance within neural cells.

## 5. Competitive Occupancy and Sensor Hierarchy in Innate Immunity Against Endogenously Formed Nucleic Acids

### 5.1. ADAR1 P150 Alters Structural Preference of RNA Prior to Its Access by Sensors

#### 5.1.1. Zα Directs Specific Sites on the RNA for Binding

When dsRNA is formed, neither the ultimate fate nor the biological function of the dsRNA is determined by RNA sequence alone. It is the relative timing of protein binding prior to formation of a competent signalling complex, as well as the geometry of protein occupancy of each dsRNA species, that determines the final biological disposition. ADAR1 p150 has a critical role in determining this fate due to its ability to incorporate several separate functional domains within one polypeptide chain: an N-terminal Zα domain, three double-stranded RNA-binding domains (dsRBDs), and a catalytic deaminase domain [115]. These architectural elements enable ADAR1 to recognize specific conformations of dsRNA, bind to long duplexes, and specifically convert certain adenosine residues to inosine. Therefore, in addition to altering the chemistry of RNA, ADAR1 has regulatory roles concerning the availability of competing sensors and the amount of time available for such sensors to bind and signal [116].

The degree to which targeting via the Zα domain is independent of catalysis was demonstrated using mice. When ADAR1 Zα-domain mutations were introduced into mice, there was an increased interferon response mediated by MDA5 in the brain despite only an approximately 20% change in total RNA-editing activity. As a result, global measurements of editing may fail to demonstrate defects in modifying small but immunologically significant RNA populations. The data suggest that the Zα domain positions ADAR1 near structurally preferred locations on duplex RNA, where the biological importance of these regions is significantly greater than their contribution to the overall editome [117].

#### 5.1.2. Spatial Distribution of Chemical Modification and Non-Catalytic Restriction of Competing Receptors

Rather than being either present or absent, chemical modification through A-to-I editing occurs at spatially defined locations across an RNA duplex. The third dsRBD of ADAR1 contains a dimerization interface that can affect the efficiency of editing at selective sites along a substrate duplex without eliminating global catalytic activity. As a result, dimerization may affect how ADAR1 binds and edits long duplexes, including whether different parts of a transcript are modified simultaneously or sequentially [6]. Therefore, a transcript can be bound by ADAR1 and be partially edited yet retain sufficient continuity to allow a second sensor to interact productively with it. The issue then becomes not simply whether inosines are present, but whether inosine incorporation results in loss of sufficient surface area to support recognition and productive assembly of a competing receptor [118].

In addition to catalytic editing, ADAR1 can also restrict RNA sensing independently of deamination. Through binding to RNA, ADAR1 can form complexes with PKR on shared duplex substrates that inhibit the kinase juxtaposition necessary for activation. This mechanism is independent of deamination-mediated regulation of MDA5. While editing tends to reduce the immunogenicity of selected MDA5 substrates, RNA binding and protein–protein interactions can suppress PKR activation regardless of whether editing has occurred completely [119,120]. Therefore, catalytic editing and non-catalytic binding provide two mechanistically distinct mechanisms for regulating competing sensors.

#### 5.1.3. Regulation of RNP Allocation and Substrate Redistribution

Regulatory actions by ADAR1 may extend beyond classical cytoplasmic self-dsRNA. Coding transcripts, elongated 3′ untranslated regions (UTRs), and mitochondria-derived transcripts exhibit increased levels of duplex RNA in cells lacking ADAR1. Additionally, ADAR1 p150 has been detected in mitochondrial preparations, whereas mitochondrial dsRNA accumulates under conditions of reduced editing capacity, suggesting the potential for ADAR1 to regulate substrate retention, processing, or turnover [19]. However, localization or detection by subcellular fractionation does not confirm intramitochondrial localization or function. As such, caution should be exercised in interpreting these findings, while they still provide evidence for the general principle that ADAR1 can modulate endogenous duplex abundance through catalytic and/or non-catalytic mechanisms [121].

Therefore, the most logical operational unit for assessing ADAR1 activity would involve the entire ribonucleoprotein complex. Zα-domain-based targeting influences which conformational states are recognized; the number and arrangement of dsRBDs influence duplex coverage and residence time; oligomerization affects the spatial distribution of editing; and catalytic or non-catalytic functions determine which competing sensors remain accessible to the substrate. Defects at any level may thus redistribute a finite substrate pool among MDA5, PKR, and ZBP1 without necessarily causing equivalent activation of all three [122,123].

### 5.2. Three Sensors for Diverse dsRNA Species

#### 5.2.1. Sensor-Specific dsRNA Substrate Populations

The dsRNA generated from RNA transcripts of the genome vastly exceeds the amount of dsRNA that will induce an autoimmune response. It was estimated that only approximately 1.1% of the dsRNA found in HEK293T cells and 2.4% of the dsRNA present in neural stem cells were subject to A-to-I editing by cytoplasmic ADAR1 to prevent interaction with MDA5. These immunogenic dsRNA species existed primarily as mature mRNA molecules containing only a few introns. Hence, they were essentially processed RNAs that had entered or were approaching the cytoplasmic compartment, where they could potentially interact with MDA5. Most of the dsRNA duplexes were formed due to the presence of inverted Alu repeats and overlapping gene products transcribed in opposing orientations [124].

Because a very low percentage of endogenously produced dsRNA can trigger an innate immune response, evaluating the total amount of self-produced dsRNA provides insufficient information regarding which specific individual species have the capacity to induce an innate immune response. Rather, the ability of an individual dsRNA species to induce an innate immune response is dependent on two parameters: (i) the inherent molecular characteristics of the dsRNA species itself and (ii) the cellular environment in which it is expressed [125]. For instance, the dsRNA species specifically produced by neural progenitor cells seem to correspond to expression programs characteristic of certain neuronal and oligodendrocytic cell populations. Conversely, dsRNA species common to many cell types probably possess equivalent levels of immunogenic potential regardless of cell type. Consequently, cell-type identity determines which subset of dsRNA species is available for binding to MDA5, whereas the molecular state of each substrate determines whether it is sensor competent [126].

#### 5.2.2. Molecular State Specificity of dsRNA Binding

MDA5 exhibits a preference for dsRNA molecules that contain long, continuous sequences capable of supporting formation of an extended filamentous receptor complex. A-to-I editing generates discrete interruptions in double-stranded RNA secondary structure and can interfere with filament formation. Nonetheless, some heavily edited candidate RNAs can also engage MDA5 under simplified biochemical conditions. Thus, loss of immunogenicity may occur through various mechanisms, including localization, protein-coat composition, and competition with other RNA-binding proteins in intact cells [127].

PKR interacts with a distinct geometric configuration of dsRNA. After PKR receptor molecules are sufficiently concentrated on a duplex such that their kinase domains are juxtaposed, eIF2α phosphorylation inhibits translation and initiates the integrated stress response. Therefore, the geometrical requirements for PKR activation need not overlap with those required for extended filamentous MDA5 assembly. Editing may render one sensor inactive while preserving a shorter or differently organized duplex surface capable of binding another sensor. Furthermore, ADAR1 can block PKR directly through RNA occupancy and protein–protein interactions, thereby dissociating PKR inhibition from deamination [128].

**Figure 2:**
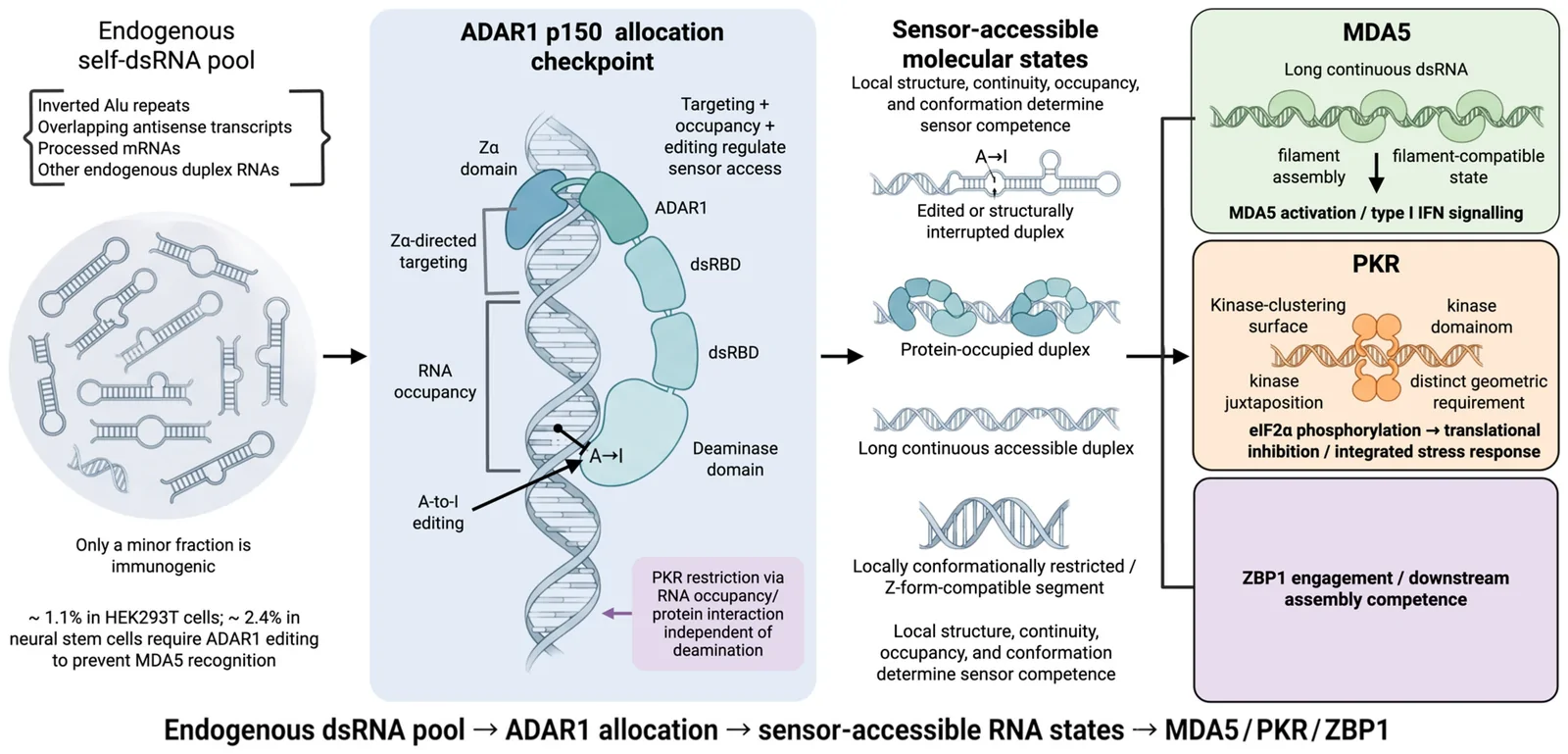
ADAR1 p150 allocates self-dsRNA before sensor engagement. ADAR1-mediated targeting, occupancy and A-to-I editing generate distinct sensor-accessible RNA states. MDA5 favors long filament-forming duplexes, PKR recognizes kinase-clustering-compatible dsRNA, and ZBP1 engages conformationally licensed Z-RNA-compatible structures.

Figure 2 places this distinction within a broader framework for dsRNA allocation, showing how ADAR1-mediated editing and occupancy transform self-dsRNA into sensor-specific states before engagement by MDA5, PKR, or ZBP1.

ZBP1 recognizes substrates based upon conformation rather than length alone. A dsRNA molecule may be large enough to activate either MDA5 or PKR yet fail to develop a stable higher-order surface capable of recruiting Zα. Alternatively, a relatively short, conformationally restricted segment embedded within a much larger transcript may develop a locus suitable for ZBP1 recognition while failing to provide sufficient filament-forming duplex for MDA5 activity. Therefore, these sensors cannot simply be viewed as competing for a common pool of dsRNA; instead, they identify highly overlapping but non-identical molecular states: filament-forming duplexes for MDA5, kinase-clustering surfaces for PKR, and conformationally licensed structures for ZBP1 [5].

#### 5.2.3. Kinetic Partitioning Among Competent Molecular States

Competition among sensors for a given dsRNA is dependent on both relative affinity and the order of occupancy. An RNA can be subjected to A-to-I editing by ADAR1 prior to filamentous assembly by MDA5; ADAR1 can occupy a duplex for sufficient time to prevent activation of PKR; and/or an RNA substrate can temporarily assume a Z-form prior to attaining sufficient conformational stability to recruit ZBP1. Additionally, prolonged conformational stability can support recruitment of ZBP1 even though significant A-to-I editing occurs throughout the majority of the remaining transcript. Therefore, local rather than global RNA structure appears essential in defining which sensor will ultimately associate with a particular RNA [129].

Therefore, an immunogenic RNA represents a dynamic set of molecular states rather than a single class of antigenic ligands. Consequently, an individual transcript can exist in multiple states: edited or unedited, protein-coated or exposed, localized in the nucleus or cytoplasm, and existing in A-form or Z-form, with varying probabilities of association with an innate immune sensor. Competition among innate immune pathways therefore arises through kinetic partitioning across an evolving landscape of ribonucleoprotein complexes [130].

Therefore, the notion of an immunogenic RNA is best interpreted as a dynamic ensemble of molecular states whose probability of interaction with a particular innate immune sensor is constantly changing due to ongoing modifications in secondary or tertiary structure, localization, post-transcriptional modification and protein coating [131].

### 5.3. Sensor Hierarchy Controls When Sensors Are Activated, How Much They Amplify Each Other’s Responses, and What Final Biological Outcome Occurs

#### 5.3.1. Interactions Between Nucleic-Acid Sensors via Signalling Networks Including Type I IFNs, Stress Responses, and Modulation of ADAR1

In addition to competing for their substrates, nucleic-acid sensors interact via signalling pathways including type I interferons (IFNs), cellular stress responses, and modulation of ADAR1. Since activation of one pathway can affect the gene expression of other sensor proteins, influence the availability of substrates for subsequent sensors, or ultimately modulate the biological outcome, the final phenotype may depend on the order in which these pathways are activated [132].

An illustrative example of the importance of this hierarchy is provided by brains from mice lacking the ADAR1 Zα domain. These brains showed signs of activation of the MDA5-, PKR-, and ZBP1-associated pathways. However, when either ZBP1 or PKR was removed, no recovery from the neurological phenotype was observed [133]. Conversely, abrogation of type I interferon signalling reversed ventricular enlargement, calcification, gliosis, white-matter damage, and loss of ependyma. Therefore, although ZBP1 and PKR were activated during the inflammatory response, neither was necessary for the major aspects of the pathological brain state. As such, evidence of biochemical or transcriptional sensor activation should not be used to infer causal significance in determining the pathological state [134].

#### 5.3.2. Secondary Recruitment of ZBP1 by Upstream Nucleic-Acid-Sensor Pathways

Further evidence demonstrating how an upstream cytosolic-DNA-sensing pathway can activate ZBP1 indirectly comes from studies using a non-neuronal bystander-cell model. Cytosolic DNA sensing through cGAS–STING led to the release of cGAMP, IFN-β, and TNF into the extracellular space. Uptake of cGAMP resulted in STING activation in adjacent cells and enhanced autophagy-dependent degradation of ADAR1, thereby allowing accumulation of Z-nucleic acids; IFN stimulated the machinery responsible for self-nucleic-acid surveillance; and TNF promoted formation of mixed inflammatory death complexes. Subsequently, secondary ZBP1-dependent apoptosis ensued in uninfected cells [135].

While this mechanism has not been shown to be applicable in neurons, it represents a mechanistic example illustrating how an upstream DNA-sensing pathway can destabilize RNA tolerance and lead to secondary recruitment of ZBP1. Collectively, these data suggest that there is a temporally ordered process. An initial sensor leads to type I interferon production; type I IFN stimulates both immune receptors and the ADAR1 p150 suppressor; subsequent prolonged cellular stress, substrate saturation, or degradation of ADAR1 leads to increased substrate accessibility to MDA5, PKR, and ZBP1; and finally, downstream effectors produce translational arrest, inflammation, or cell death depending on the unresolved state. Because type I IFN increases both nucleic-acid-surveillance mechanisms and the ADAR1 p150 suppressor, this network is inherently nonlinear [136].

Therefore, disease progression in neural tissues may be due to alterations in sensor order rather than the emergence of new ligands. Early type I IFN signalling may promote ADAR1-mediated tolerance while concurrently preparing MDA5, PKR, and ZBP1 for rapid antimicrobial responses. However, once transcriptional, mitochondrial, or proteostatic stresses persistently disrupt editing or occupancy capacity, additional sensors may gain access to substrates that were previously protected by ADAR1. Therefore, what initially appears as a secondary indicator of an inflammatory state may eventually evolve into a causal amplifier as the molecular environment changes [137].

#### 5.3.3. Experimental Dissection of Initiating Sensor(s), Amplification Pathway(s), and Terminal Effector(s)

Therefore, deconstruction of this mechanism necessitates distinction among at least three orders of action: (1) the initiating sensor(s); (2) the pathway(s) responsible for signal amplification; and (3) the terminal effector(s). Data collected at only a single time point, e.g., accumulation of ligands, induction of receptors, or markers indicative of cell death, cannot accurately reconstruct the temporal sequence among these actions.

Genetic interaction and epistasis studies, acute protein depletion, substrate-resolved mapping, and long-term single-cell analyses are needed to define whether ZBP1 initiates the pathological state, enhances an MDA5-driven interferon programme, or acts downstream of cGAS–STING-mediated disruption of RNA tolerance [22].

Temporal control over perturbations combined with cell-type-specific manipulations and substrate-resolved measurements within the same experiment represents the most robust design for assigning causality to each action within the sensor hierarchy. Early inhibition before pathology development would support an initiating role for ZBP1; delayed inhibition that reduces progression even though the initiating lesion remains present would support an amplifying role for ZBP1; and interruption of ZBP1-dependent downstream effector activation after upstream signalling has already occurred would support a terminal execution role. These studies will differentiate pathway presence from pathway necessity and provide a more rigorous framework for defining causal position within the sensor hierarchy. Therefore, competitive occupancy of nucleic acids by sensors, the state of the nucleic-acid substrate, and the temporal ordering of sensor engagement govern endogenous nucleic-acid surveillance. This hierarchical organization explains why the same set of induced receptors can correspond to different biological outcomes and why identification of pathway activation does not establish causal dominance [138]. Section 6 translates this network logic into human molecular endotypes, wherein ligand identity, sensing cell, receptor complex and terminal effector must be identified within the same disease context.

## 6. Human Causal Endotypes and Translational Reconstruction

### 6.1. Endotypes Represent Ordered Causal Architectures; They Do Not Represent Collections of Biomarkers

#### 6.1.1. Reconstruction of the Causal Sequence Using Cellular Resolution

An endotype for ZBP1 in humans cannot be determined exclusively based on the amount of ZBP1 expressed in target cells or the production of type I interferons after ZBP1 signalling has been activated. These data demonstrate the presence of biochemical pathways involved in the measured activity; however, they do not define the temporal sequence or the relative necessity of each biochemical event within a particular endotype [139]. Therefore, in this review, we refer to the interpretative model used here to reconstruct an endotype as a causal model. This model requires specification and linkage of the following five biochemical elements of a causal chain: (i) the initial nucleic-acid lesion; (ii) the cell that generates the ligand; (iii) the cell that expresses ZBP1 and senses the ligand; (iv) the formed receptor–adaptor complex; and (v) the final biological effect. Additionally, this model incorporates time, since the predominant mechanism(s) contributing to a specific endotype can change as ligands are continuously generated by ligand-producing cells, interferon tone continues to increase and receptors become available or unavailable [140].

Therefore, this model describes an endotype as a causal relationship among biochemical pathways and not merely as a collection of molecules associated with disease. For example, a cell-autonomous circuit indicates that there is no distinction between the cell in which the ligand is produced, the cell that senses the ligand through ZBP1, and the cell in which execution takes place. In contrast, a transcellular circuit indicates that one cell population produces or exports nucleic acids, another cell population is primarily responsible for detecting those nucleic acids through ZBP1, and potentially a third population experiences the ultimate effects due to interactions within the tissue environment [141]. Since these configurations could result in similar overall biochemical readouts, they would likely require distinctly different therapeutic approaches. Thus, elevated levels of ZBP1 protein expression in neurons, increased activation of microglia, and phosphorylation of death-associated proteins in adjacent areas of a tissue section do not demonstrate a single causal pathway unless evidence is provided to support their spatial and temporal relationships [90].

#### 6.1.2. Activation of Pathways Versus Causal Dominance

Thus, it is necessary to differentiate between pathway activation and causal dominance. There was significant activation of multiple nucleic-acid-responsive pathways in the brains of mice deficient in the ADAR1 Zα domain; however, deficiency of either ZBP1 or PKR did not alleviate the observed neurological defects. Conversely, blocking type I interferon signalling alleviated ventricular enlargement, calcification, gliosis, white-matter degeneration, and loss of ependymal cells in these mice, suggesting that type I interferon signalling was the predominant disease-sustaining mechanism in that experimental setting [60]. Moreover, although MDA5-dependent activation of brain interferon signalling was strongly induced by ADAR1 Zα-domain dysfunction, global RNA-editing measurements showed only minimal alterations, thereby demonstrating that, even when bulk molecular measurements appear relatively unchanged, a very small subset of immunogenically potent nucleic acids can produce disproportionately large biological effects. Thus, human endotypes need to be constructed hierarchically through perturbation rather than merely inferred from the abundance of biochemical pathways [124].

The initiating element is the earliest lesion whose selective correction prevents downstream pathway formation. The amplifying element supports or continues the response initiated by the first lesion. The executing element directly produces inflammatory transcription, translational arrest, or cellular damage. Importantly, the same receptor may have different roles at different stages of disease. Thus, ZBP1 may initially represent an IFN-induced marker of pathway competence following nucleic-acid lesions; later in disease progression, ZBP1 may become an amplifier as ligand concentration increases; and eventually, ZBP1 may participate in an execution complex once sufficient receptor assembly has occurred [142].

#### 6.1.3. Temporal Plasticity of Human Endotypes

Temporal plasticity is especially relevant in chronic neurodegenerative diseases. A fixed biopsy or post-mortem sample may include residual products from several stages of disease progression. Thus, determination of which molecular dependency is active at the time of sampling should be considered in addition to determining which molecules are present. An individual patient’s clinical diagnosis may include ZBP1 circuits dominated by initiators in some cells or anatomical regions and circuits dominated by amplifiers or executors in other cells or anatomical regions [143].

Therefore, the appropriate translational unit is the ligand–sensor–complex–effector chain resolved at the cellular level. Clinical diagnoses provide critical clinical information but, by themselves, do not identify the biochemical architecture responsible for disease progression [144].

### 6.2. Proteoform Stoichiometry Defines Receptor Competence

The measurement of overall ZBP1 quantity does not fully represent signalling capability, since alternative splicing could decouple Z-nucleic-acid binding from the triggering of signal execution. A short murine isoform (ZBP1-S), which has functional Zα domains but lacks the complete signalling apparatus of full-length ZBP1, competes with full-length ZBP1 for Z-nucleic-acid binding and blocks the activation of RIPK3-, MLKL- and caspase-dependent signalling pathways mediated by full-length ZBP1. This short isoform was also shown to be more readily induced by interferon and more susceptible to proteasomal degradation than the longer isoform, suggesting that the inhibition it exerts on the signalling activity of full-length ZBP1 would likely occur rapidly and transiently [145].

A novel stoichiometric checkpoint has thus been identified. The amount of ligand bound does not directly equate to the amount of signalling produced because the same structural substrate can be bound by either an execution-competent or a recognition-only proteoform. Therefore, a tissue containing a large amount of total ZBP1 may fail to respond to ligand even though it contains ample amounts of receptor [146]. Conversely, a tissue with a low level of receptor may become activated upon exposure to the same ligand if there is a significant decrease in the relative proportion of recognition-only forms that would otherwise capture most of the ligand. Since the final output of the signalling pathways examined here occurs through a cooperative process, small shifts in the relative proportions of different proteoforms could cause the system to cross an “execution” threshold [147].

Standard methods employed in molecular biology for measuring mRNA or protein levels within tissues, including short-read sequencing, quantitative RT-PCR and Western blot analysis, are not necessarily reliable for distinguishing these states. The reason is as follows: short-read sequencing may group transcripts containing similar exon structures together, making it difficult to distinguish between different transcripts encoded by the same gene. Antibodies that recognise amino-terminal sequences conserved among receptor proteoforms may bind equally well to all forms, regardless of whether they possess the ability to activate a signalosome. Therefore, total ZBP1 mRNA, pan-ZBP1 immunoreactivity and conventional single-cell expression profiles may substantially overestimate the number of receptors present per cell that are capable of assembling into a productive signalosome [148].

Although multiple human ZBP1 transcripts have been documented, most lack detailed information regarding their translation, stability and cell-specific abundance in human neural tissues. Predicted ZBP1 isoforms should therefore be considered unvalidated until further studies demonstrate their existence at the protein level and provide evidence that they are present in neural tissues. Studies performed in heterologous systems cannot by themselves establish that such isoforms are relevant to a particular neural cell type [149].

To determine whether any given isoform represents a receptor-competent form of ZBP1, it will be necessary to identify the possible ZBP1 mRNA isoforms in each neural cell type studied using approaches such as long-range PCR, cDNA cloning and long-read transcript analysis. Each isoform must then be analysed to confirm its translated product and subsequent subcellular localisation. Furthermore, receptor-competent forms of ZBP1 should be examined using antibodies directed against sequences unique to each form and through functional assessment of their ability to assemble with molecules involved in ZBP1-initiated signal-transduction cascades [150].

In addition to differences in primary sequence among species, another factor contributing to variability in downstream pathway assembly is compatibility among adaptor molecules. For example, in interferon-primed human HT-29 cells, fibroblasts and U937 monocytes, ZBP1-driven necroptosis required RIPK1 for stable association of ZBP1 with RIPK3, RIPK3 activation and MLKL phosphorylation [151]. In contrast, no obligatory role for RIPK1 was observed in the murine equivalents. Replacement of the human RIPK3 RHIM interface with its murine homologue restored RIPK1-independent RIPK3 activation and MLKL phosphorylation, demonstrating that differential use of adaptor interfaces between species can fundamentally alter the organisation of an otherwise highly conserved signalling pathway [152].

Therefore, murine models used to delete genes encoding components of the ZBP1 signalling pathway do so in a context in which ZBP1 can associate directly with RIPK3 without requiring an additional adaptor. Thus, deletion experiments performed in mice do not by themselves define the signalling-competent receptor complex in humans. Rather than relying simply on the fact that many human genes share names with murine orthologues, what matters is compatibility among the human receptor proteoform, its RHIM interfaces and the locally available adaptors [84].

Thus, in humans, signalling competence is determined by the overlap of three variables: the fraction of ligands occupied by full-length ZBP1; the abundance and stability of forms that recognise ligands but do not execute signalling; and the availability of compatible partner proteins for downstream assembly. None of these factors can be estimated by considering total receptor expression alone [153].

### 6.3. Human Causality Requires Same-Lesion Molecular Concordance

A human endotype must demonstrate molecular correlation, including ligand conformation and receptor occupancy, as well as spatial correlation with the site of injury. Molecular correlation is based upon the principle of structure–occupancy–complex–consequence concordance; this principle states that, for a given disease process, there must be agreement among ligand architecture, receptor occupancy, assembly of the receptor–adaptor complex, and the ultimate consequence of that interaction [154]. The structural aspect defines the chemical nature of the ligand, which determines how it is able to interact with the receptor. This includes identifying the individual molecules present, reconstructing their complete structure, determining whether chemical modifications have occurred, establishing whether they consist of single-stranded or double-stranded nucleic acids, and identifying where in the cell they originated. Long-read sequencing methods are particularly important when competence depends upon long stretches of 3′ untranslated sequences, retained introns, chimeric mRNAs, or widely spaced inverted repeats [155].

Occupancy refers to the physical binding of ZBP1 to its native endogenous ligands. It is essential that a conformation-selective capture method identifies the same molecule used to establish receptor occupancy. Additionally, alteration of the Zα-domain interaction surface should significantly decrease the amount of ligand bound without affecting the total number of receptors. Furthermore, the specific ZBP1 proteoforms captured must be identified. Binding of ZBP1 to a ligand using a truncated protein containing only the Zα domain does not provide evidence that such binding results in a productive signaling event [111].

Complex refers to the assemblies that occur subsequent to establishment of the initial ligand–receptor interaction. Techniques such as native-complex proteomics, proximity labeling, and endogenous co-capture will help determine whether ZBP1 interacts directly with RIPK1, RIPK3, caspases, or other proteins. Although detergent-insoluble complexes and punctate structures suggest higher-order organization, neither provides independent evidence for the presence of a functional RHIM polymer or its associated adaptor proteins [156].

Consequences refer to the downstream effects of the interactions established between the ligand and receptor. Such consequences include RIPK3 activation, MLKL phosphorylation and oligomerization, followed by membrane redistribution. To assign a necroptotic response, it is necessary to document these events within the same relevant cell type and establish their functional relationship. Establishing a RIPK1-mediated inflammatory response requires demonstration of RIPK1 activity associated with transcriptional changes without assuming cytotoxicity [104]. Similarly, establishing an apoptotic response requires documentation of caspase activity coupled to the appropriate scaffolding proteins. Spatial separation of ligand generation, receptor localization, and membrane injury is informative about the mechanisms involved in signal transduction rather than being simply an artifact of sample collection. For example, if a neuron generates a ligand that leads to microglial assembly of a receptor complex and subsequent membrane injury occurs in adjacent cells, this would suggest the existence of a transcellular circuit, provided that the route taken and sequence of events can be established. In the absence of such information, the data would represent separate concurrent tissue responses rather than a single causal endotype [157].

This framework for assessing causal relationships will likely affect biomarker development. Circulating mtDNA, extracellular-vesicle cargo, cytokine and chemokine profiles, repetitive RNA sequences, and ZBP1 mRNA expression levels are individually nonspecific. When examined collectively as part of an endotype-resolved profile—that is, defined ligand architecture or oxidative state, proteoform-specific ZBP1 occupancy, compatible adaptor recruitment, and a consistent terminal-effector phenotype—their specificity may improve substantially. Therefore, future biomarkers should aim to measure molecular organization rather than individual components [158].

Ultimately, human ZBP1-related diseases involve problems of causal ordering. A particular receptor density can be associated with different outcomes depending on the corresponding proteoforms, adaptor compatibility, cell types involved, and disease stage. Thus, translation requires reconstruction of this ordering at physiological concentrations and, ideally, within the confines of a single lesion [139]. Table 3 provides a comparative synthesis of the principal ZBP1-associated disease and experimental endotypes, integrating ligand identity, cellular context, downstream signalling, evidentiary strength, human validation and therapeutic implications.

## 7. Experimental Reconstruction and Endotype-Matched Interception of ZBP1 Pathology

### 7.1. Establishing an Authentic Endogenous ZBP1 Ligand

The principal experimental question is to determine whether a given nucleic acid is merely present at concentrations high enough to be considered “abundant,” is “repetitive,” or has a secondary structure of some type, or whether it can form a complex with endogenous ZBP1 and is thereby capable of initiating signaling through ZBP1. While no single assay can provide sufficient information to identify an authentic endogenous ligand, evidence from multiple assays supporting all five criteria will provide a reasonable basis for assigning a particular nucleic acid as a specific ZBP1 ligand [163]. Specifically, the criteria include evidence for the chemical composition of the nucleic acid, evidence for its native-state structure, evidence for its molecular structure—particularly whether it contains sequences or structures capable of being recognized by ZBP1—evidence for association of the nucleic acid with ZBP1 (occupancy), and evidence that modification of the identified nucleic acid results in loss of ZBP1 activation [5].

Evidence for chemical composition can be established using enzymatic treatments, including controlled treatment with DNase, duplex-selective RNase, single-strand-selective RNase, and RNase H. These treatments can selectively digest DNA, RNA–RNA duplexes, single-stranded RNA, and RNA:DNA hybrids, respectively. However, each enzyme must be evaluated to ensure that digestion occurs efficiently and preserves native protein–nucleic-acid complexes. Similarly, generic dsRNA or cytosolic DNA staining can identify a large population of potential substrates but provides little information regarding the specific molecular species interacting with ZBP1 [164].

Support for native-state structure can be obtained using additional orthogonal methods. For example, antibodies that selectively recognize Z-forms of DNA or recombinant Zα-based probes have been developed to preferentially bind regions of a nucleic acid that adopt a Z-form. However, both types of probe have inherent biases because they may preferentially stabilize nucleic-acid conformations that are only transiently formed in vivo [165]. Therefore, control experiments lacking the probe, mutations in Zα that disrupt binding, competition experiments with wild-type or mutant probes, and nuclease resolution of bound versus unbound nucleic acids must be performed. Agreement between structurally distinct capture reagents is far more informative than simply comparing the levels of signal generated by a single reagent [166].

Evidence for molecular structure should be obtained at the level of the entire molecule and not merely at the nearest annotated locus. Short-read sequencing technologies have proven useful in identifying enrichment of a specific genomic region; however, these technologies do not always allow accurate reconstruction of long 3′ extensions of mRNAs, retained intronic sequences, chimeras consisting of host and repeat sequences, bidirectional transcription, or distant inverted repeats contained within a single molecule [167]. In contrast, long-read sequencing technologies, together with strand-specific mapping and junction-resolved amplification techniques, have the added benefit of resolving long-range connections between different parts of a transcript that are necessary for establishing conformational compatibility. Resolution of fragment boundaries, oxidation states, strandedness, and subcellular localization is similarly critical when identifying candidate mitochondrial molecules [168].

Association with ZBP1 can be determined directly by cross-linking and capturing endogenous ZBP1 and demonstrating that the recovered substrate is identical to that identified by the conformational assay. Moreover, disrupting the Zα–ligand interaction surface should prevent binding without affecting either the overall amount or localization of ZBP1. Colocalization alone is insufficient because, in interferon-rich environments, both ZBP1 and aberrant nucleic acids can colocalize without participating in a physical interaction. Finally, proteoform identity should also be established, since occupancy by a recognition-only isoform does not demonstrate formation of a signaling-competent receptor [169].

Ultimately, the most compelling evidence for causality in this system will come from modifying the ligand itself. For example, restoration of an aberrant termination site, repair of a splicing defect, removal of one member of an inverted-repeat pair, or prevention of mitochondrial oxidation should result in reduced ZBP1 occupancy and subsequent signaling while preserving as much of the surrounding gene or organelle as possible. If re-expression of the corrected substrate restores signaling, whereas expression of a sequence-matched substrate incapable of adopting the correct topology does not, causality is further reinforced. Simply transfecting cells with cDNA encoding the putative ligand would not provide conclusive evidence for its role as an endogenous ligand, because such transfection alters concentration, compartmentalization, protein coating, and uptake pathways simultaneously [170].

**Figure 3:**
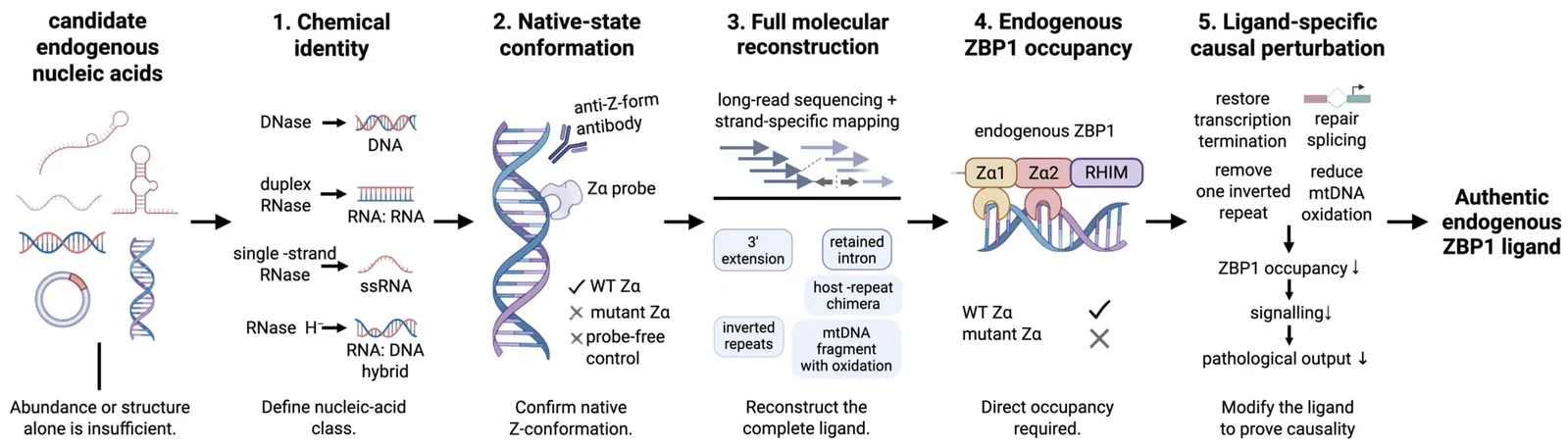
Experimental reconstruction of an authentic endogenous ZBP1 ligand. Candidate nucleic acids are progressively validated through five criteria: chemical identity, native-state Z-conformation, full molecular reconstruction, Zα-dependent endogenous ZBP1 occupancy, and substrate-specific causal perturbation. Convergence of all five criteria distinguishes structurally compatible nucleic acids from authentic endogenous ZBP1 ligands.

Therefore, we propose that an authentic endogenous ligand must meet a five-point standard: nucleic-acid-resolved chemical identity, native-state conformational evidence, full molecular reconstruction, dependence upon Zα-mediated ZBP1 occupancy, and substrate-specific causal perturbation. This standard intentionally exceeds the evidentiary threshold associated with detecting Z-form-related material. Our proposed standard transforms structural inference into a falsifiable molecular mechanism [171]. Figure 3 summarizes this five-point experimental framework, integrating chemical identity, native-state conformation, full molecular reconstruction, endogenous ZBP1 occupancy, and substrate-specific causal perturbation. Together, these criteria distinguish structurally compatible nucleic acids from authentic endogenous ZBP1 ligands.

### 7.2. Reconstructing Pathway Order in Human Neural Systems

Identifying a ligand is not sufficient to determine when ZBP1 first produces observable effects. Typically, the final stage of this process involves the coexistence of all known intermediate signs, including death-pathway indicators, receptor clusters, interferon responses, and nucleic-acid stress. Therefore, to distinguish early, middle, and late phases of progression, the temporal sequence must be determined [5].

Human induced pluripotent stem cells (hiPSCs) can produce representative models of neurons, microglia, and astrocytes. However, a monotypic culture of neural cells alone cannot be used to differentiate ligand transfer and/or paracrine signaling. Mixed cultures of neural cells, such as assembloids, organoids, and related systems, permit independent modulation of populations producing the ligands, detecting them, and experiencing their terminal effects. As an example, a neuronal candidate ligand could be tested alongside selective deletion of ZBP1 in either neurons or microglia and selective inhibition of nucleic-acid export or vesicle-mediated ligand transport [172].

These levels of intervention represent distinct mechanisms for intervening in the processes under investigation. Upstream interference disrupts ligand synthesis while leaving intact the cellular apparatus capable of sensing ligands. Recognition-level interference alters particular Zα surface sites that contact the ligand while leaving the RHIM-containing signaling scaffold intact. Execution-level interference alters the interfaces of individual adaptor molecules within the signaling complex or terminates signaling without interfering with ligand binding to its receptor. Consistency across all three types of interference indicates an ordered flow of activity along a pathway. Inconsistencies among the three indicate additional mechanisms, such as multiple sensors, scaffold-independent signaling, or secondary amplification [68].

Temporal regulation is generally preferable to constitutive knockout of ZBP1, RIPK1, or RIPK3 when establishing the order in which pathway components operate. Each of these proteins contributes independently to different functions, including neuronal differentiation, basal IFN tone, and compensatory sensor expression. Inducible degrons, targeted proteolytic degradation, and temporally controlled genome modification permit disruption of a protein after neural maturation, after appearance of the ligand, or after formation of the ligand–receptor complex. Such studies would then determine whether ZBP1 initiates disease pathology, stabilizes an existing signaling complex, or becomes unnecessary once downstream amplification has occurred [73].

Maintaining endogenous stoichiometry is essential. Excessive receptor expression may bypass the requirement for recognition and modify selection among adaptor molecules; heterologous rescue using incompatible RHIM domains can also be problematic. Therefore, endogenous tagging and knock-in mutations expressed at physiological levels provide better options than transgenic overexpression. Furthermore, when evaluating assembly inhibitors, it is important to preserve the ability of RIPK1 to stabilize the ZBP1–RIPK3 complex in a RIPK1-dependent manner consistent with what has been demonstrated in human cells [173].

A causal relationship requires defining a sequence of four temporally ordered events: appearance of the candidate ligand, Zα-dependent occupancy of ZBP1, assembly of a functional adaptor complex, and activation of a terminal effector. Longitudinal imaging, proximity-based mapping of complexes, native proteomics, single-cell multi-omics, and proteoform-resolved sequencing can be employed to assess whether these events occur sequentially within one cell type or are propagated through a producer–sensor circuit [174].

Thus, the optimal experimental paradigm does not eliminate the entire pathway before cellular development begins, but instead identifies a specific molecular event occurring at a specific time point. The causal perturbation ladder will transform static associations among pathway components into a logical and testable sequence of events [175].

### 7.3. Therapeutic Intervention Should Match Causal Depth and Disease Phase

ZBP1-associated pathology, such as aberrant microglial activation, can be prevented at the level of ligand production, Zα-mediated ligand recognition, ZBP1 receptor stability, ZBP1 signalosome assembly, or execution downstream of the ZBP1 signalosome. Each of these interventions occurs at a distinct causal depth and is therefore not equivalent [90].

Intervention at the level of the source directly addresses mechanisms most proximal to the causal defect. For example, restoration of normal regulation of transcription termination, splicing of RNA precursors by the spliceosome, heterochromatic regulation of gene expression, or reduction in mitochondrial oxidative damage may prevent multiple sensors from being exposed to the same anomalous substrate. Such strategies may effectively reduce stress responses to nucleic-acid abnormalities broadly throughout the organism; however, they may also disrupt critical transcriptional, post-transcriptional, and organelle functions, thereby limiting their utility [176].

Blocking receptor recognition of pathological substrates is likely to provide a higher degree of receptor specificity. Shorter forms of ZBP1 exist naturally, such as ZBP1-S. Although ZBP1-S retains functional Zα domains, it lacks the complete signaling apparatus of full-length ZBP1 and competes with full-length ZBP1 for structurally compatible substrates [88]. This provides a conceptual model for developing non-signaling “decoy” molecules that inhibit ZBP1 recognition of specific substrates by binding to those substrates competitively. Similarly, constrained analogs of Zα or compounds that occlude the ZBP1 surfaces mediating substrate binding may be developed. However, such molecules will need to be designed so that they selectively bind pathological substrates without broadly sequestering physiologically important nucleic acids [177].

Another potential site for intervention is ZBP1 receptor proteostasis. In microglia subjected to severe injury, the stability of ZBP1 is maintained through USP4-mediated deubiquitination. Pharmacological inhibition of USP4 results in degradation of ZBP1 and decreased inflammatory death of activated microglia. Reduction in receptor half-life could potentially prevent cooperative assembly of the ZBP1 receptor complex while maintaining some degree of basal surveillance [93]. However, the major limitation of this approach is specificity, since enzymes involved in ubiquitin modification regulate multiple substrates and would likely require either targeted delivery to the cells in which they are needed or specific disruption of the interaction between ZBP1 and USP4 rather than general inhibition of the deubiquitinating enzyme [178].

Inhibition at the level of assembly is particularly relevant given the recent identification of components of the human ZBP1 receptor complex. Pharmacological inhibition of RIPK1 can prevent assembly of the RIPK1-containing ZBP1 receptor complex in human cell-based models and decrease inflammatory death of activated microglia in experimental models of amyloid-associated disease. However, the effects of RIPK1 inhibition will depend upon the functions of RIPK1 within the relevant endotype. Inhibiting RIPK1 kinase activity may prevent inflammatory or necroptotic signaling while preserving scaffold-dependent functions, including apoptosis; disrupting the interaction between the RHIM interfaces of RIPK1 and ZBP1 may produce a different outcome. Therefore, designing RIPK1-based therapies will require distinguishing its enzymatic activities from its structural roles within the signalosome [73].

Pharmacological modulation of ZBP1 itself remains at an early stage. Compounds capable of binding ZBP1 and decreasing receptor-associated inflammatory death outside the central nervous system demonstrate that the receptor is druggable; translation into the CNS will require demonstration of binding to human proteoforms, passage across the blood–brain barrier, selectivity relative to other nucleic-acid-binding proteins, and preservation of antiviral defense [5].

Modulation at the level of terminal effectors is likely to provide rapid protection against cell death but will occur furthest from the initial insult. Blockade of RIPK3 or MLKL is logical only if necroptotic execution has been demonstrated. The use of caspase inhibitors may redirect signaling towards necroptosis rather than eliminating it entirely; the use of IFNAR blockers is justified only if IFN-driven positive-feedback loops represent the predominant disease-maintenance mechanism. Mixed programs of cell-death execution may therefore require combinations that simultaneously suppress parallel or compensatory branches without enhancing alternative modes of cell death [179].

Time is as important as target choice. Microglial ZBP1 activity early after injury may contribute to containment or recovery, whereas continued activity may promote sustained inflammation or depletion of sensor cells. Continuous pathway blockade may therefore be ineffective—or even detrimental—compared with phased or temporally restricted interventions. Identification of both the endotype and its position along the causal sequence using appropriate biomarkers is therefore required [180].

The therapeutic principle is one of causal-depth matching. Defined defects in the generation of a specific ligand favor source-level correction; defective capture of a pathological ligand favors recognition blockade; stabilized receptor levels favor proteostatic intervention; human RIPK1-dependent assembly favors assembly-level inhibition; and documented terminal execution favors effector inhibition. ZBP1 abundance does not predict which strategy should be used [181].

Thus, convergence between the requirements for experimental proof and therapeutic design emphasizes the necessity of selecting an intervention that targets the molecular transition identified as causal in the appropriate cell type, species, and disease phase. This paradigm ultimately directs attention to how structural failures in nucleic-acid homeostasis give rise to decisions encoded in molecular assemblies and why this transition may create new boundaries for innate immune discrimination within the nervous system.

## 8. Conclusions

ZBP1 is increasingly recognized as a “molecular reader” of structural damage to self-nucleic acids. Activation of ZBP1 is not determined solely by the presence, quantity, or cellular context of self-nucleic acids. It is also influenced by their structural integrity, the chemical modifications they have undergone, their association with other proteins, their displacement from normal sites of localization and the amount of time for which they remain structurally stable.

When normal chromatin structure, RNA processing or editing, or mitochondrial function is disrupted, self-nucleic acids that were previously protected or compartmentalized become exposed to surveillance. This creates the possibility that they will elicit an immune response because structural abnormalities have caused them to lose their functional identity and acquire properties of non-self.

Therefore, a more modern perspective on the concept of self–non-self has emerged. Self-derived structures can elicit an immune response without being foreign. They may instead fail in a manner that causes them to lose their functional identity through structural abnormalities that prevent normal processing, shielding, or compartmentalization. From this perspective, the role of the innate immune system is not only to recognize molecules that are foreign, but also to identify molecules that have lost their functional identity. Both the nature of the damaged molecule and the length of time for which it remains structurally abnormal will influence whether an immune response occurs. For example, mitochondrial fragments containing oxidative damage provide evidence that mitochondria are damaged and that their contents have escaped from their normal membrane-bound compartments. Therefore, each form of damaged self-nucleic acid possesses structural “provenance.” This means that each form contains physical evidence of the biochemical process that failed.

Although structural provenance may identify the origin of damaged self-nucleic acids, it is not sufficient to activate signaling. The damaged self-nucleic acid must first adopt a conformation that permits productive binding to the Zα domains of ZBP1. Once this Zα-domain interaction occurs, ZBP1 converts the geometric characteristics of the damaged self-nucleic acid into receptor density and orientation. Receptor assembly through RHIM domains then produces cooperative interactions that recruit kinases, scaffold proteins, and terminal effectors. Therefore, the greatest amplification step in this signaling pathway is the conversion of a localized and potentially transient conformational event—ligand binding—into a cooperative protein complex capable of generating large-scale signaling events.

Moreover, this conversion process is inherently nonlinear. A damaged self-nucleic-acid ligand may be present at concentrations far greater than those required for detection and still fail to generate an observable response if its affinity for ZBP1 is low, receptor concentration is insufficient, ZBP1 primarily exists as a nonproductive proteoform, or the ligand remains structurally competent for too short a period to support receptor assembly. Conversely, small increases in receptor concentration, the proportion of signaling-competent ZBP1 proteoforms, or the duration for which the ligand remains structurally competent can facilitate receptor assembly. ZBP1 proteoforms that do not support productive receptor assembly can buffer this transition by competing with signaling-competent proteoforms for the same structural substrates. Stabilization of the receptor through post-translational modification or interferon-mediated upregulation can instead promote productive assembly. Therefore, although measuring total ZBP1 abundance can provide useful information, it cannot define the mechanistic significance of the receptor without additional data regarding ligand occupancy, receptor-proteoform composition, and adaptor compatibility.

In addition, the final assembly state of the ZBP1 receptor complex does not provide an unambiguous prediction of its functional outcome. Although human and murine ZBP1 systems share similarities in RHIM organization, their dependence on RIPK1 and the architecture of downstream signaling complexes can differ. The downstream programs generated by ZBP1 signaling also depend on the responding cell type. In microglia, ZBP1 signaling can support transient surveillance, sustained inflammatory transcriptional programs, or cell death. In neurons, it can lead to irreversible structural degeneration. Furthermore, nucleic-acid transfer between cells separates the ligand-producing cell from the sensing cell and therefore enables ZBP1 activity to participate in distributed producer–sensor–effector networks.

Thus, defining a single mechanism of action for ZBP1 across an entire category of neurological diseases is not reasonable. Many diseases may contain multiple spatially and temporally distinct endotypes characterized by cell-autonomous neuronal execution, microglial inflammatory sensing, secondary activation within an interferon-dominant state, or transcellular transmission of nucleic-acid-induced stress. Therefore, the correct unit of analysis for studying diseases involving ZBP1 activity is the ordered chain linking ligand origin, receptor occupancy, signalosome composition, and biological outcome.

Similarly, this ordered chain defines the basis for establishing a mechanistically supported explanation for any given endotype. Increased ZBP1 expression, staining associated with Z-DNA or Z-RNA indicative of aberrant self-nucleic-acid conformations, the presence of mtDNA outside mitochondria, repetitive RNA sequences associated with transcription-termination failure, or RNA:DNA hybrids formed during defective processing or splicing do not independently justify conclusions regarding mechanism. An identifiable mechanism for a given endotype requires demonstration that all elements within the ordered chain are mutually consistent: the structural identity of the self-nucleic acid involved, the level of Zα-dependent ZBP1 occupancy, adaptor-complex formation, recruitment and activation of terminal effectors, and spatial linkage within the cellular network. Reconstruction experiments must therefore preserve the stoichiometric relationships observed in vivo and distinguish initiating events occurring early in the signaling cascade from later amplification and execution events.

Similar reasoning applies to the development of treatments for diseases involving ZBP1 activity. Treatment strategies based solely on receptor abundance may misidentify the phase during which a ZBP1-associated pathological mechanism is active and may suppress potentially adaptive or protective responses. Identifying therapies using endotype-specific biomarkers will therefore be critical for achieving therapeutic specificity. These biomarkers should provide information regarding the operative ligand, receptor-proteoform status, the architecture of assembled adaptor complexes, and the stage of disease progression.

Despite recent advances in our understanding of ZBP1 signaling pathways, several fundamental questions remain unanswered. No comprehensive map of endogenous ZBP1 ligands in the human brain is available, little is known about ZBP1 proteoform composition, and the point along the signaling pathway at which receptor assembly becomes irreversible has not been established. Whether ZBP1 preferentially recognizes pre-existing Z-form conformers or stabilizes otherwise transient structural states remains unknown. It is also unclear whether successive sensors can use the same structural substrates and whether signalosomes remain capable of transmitting signals after losing contact with their original ligands.

Each of these questions can now be addressed using emerging methodologies, including native-state structural-capture techniques, long-read sequencing technologies for ligand reconstruction, proteomic analysis of endogenously assembled complexes, acute cell-restricted perturbation studies, and spatially resolved methods for analysing human tissues.

This framework not only contributes to the continuing debate over what defines innate immune identity, but also indicates that such identity is not determined exclusively by molecular origin. Instead, it is continually shaped by conformational state, local environment, and competitive interactions among molecular entities. Ultimately, insights provided by ZBP1 will contribute to our understanding of how cells recognize structural failures arising from their own dysfunction and translate those failures into decisions regarding adaptation, inflammation, survival or death.

## Figures and Tables

**Table 1:** Shows evidence from structural biology, transcriptomics, RNA processing, chromatin biology, mitochondrial stress and innate immune signaling to illustrate how sequence composition, duplex topology, chemical modifications, subcellular trafficking, and receptor avidity collectively determine whether an endogenous nucleic acid progresses from a Z-form-compatible intermediate to a functionally validated ZBP1 ligand.

Licensing Axis	Molecular Architecture	Quantitative or Structural Anchor	Competence Gained	Decisive Validation	Reference
Conformational predisposition	Alternating purine–pyrimidine tracts; CG-rich duplexes; syn–anti glycosidic alternation; negative supercoiling	Z-DNA: ~12 bp per left-handed turn; ~18 Å diameter	Lower energetic barrier to persistent Z-state formation	NMR, crystallography, circular dichroism, topology-controlled assays	[52]
Chemical and surveillance tuning	5-methylcytosine; 8-oxoguanine; A-to-I editing; ADAR1p150 Zα binding	A → I editing in ~68.3% of mapped host Z-RNAs; ADAR1 Zα variants P193A/P195A and W197A impair restraint	Duplex stabilization, remodeling or failure of ligand suppression	Modification-resolved sequencing; Adar1–Zbp1 epistasis	[53]
Duplex continuity and avidity	Long dsRNA, dsDNA or RNA–DNA hybrids; repeated accessible Z-surfaces	Tandem Zα1–Zα2 engagement; ligand-length- and concentration-dependent ZBP1 self-association	Transient binding → stable receptor clustering	Duplex-length titration; Zα mutants; oligomerization assays	[54]
Termination-driven transcript extension	CPSF failure; RNA polymerase II readthrough; downstream LINE, SINE and LTR capture	1646 murine and 1818 human Z-RNAs; ~78% of murine substrates from extended 3′ regions	Distant repeats become covalently linked within one RNA	Long-read reconstruction; CPSF perturbation and rescue	[55]
Repeat-pair topology	Inverted LINE1, B1/B2 SINE, Alu, ERV or LTR elements	~88% of mapped murine Z-RNAs contained inverted EREs; human genome contains >10^6^ Alu copies	Kilobase-scale fold-back duplex formation	Strand orientation, repeat-pair mapping, targeted deletion	[56]
Extreme transcript architecture	Long host–repeat chimeras and cryptic bidirectional transcripts	Nabp1 extension ~45 kb, ~16-fold longer; LINE1 pairs separated by ~1.8 and 8.8 kb	Motif-scale propensity → transcript-scale ligand architecture	Isoform reconstruction; Z22 enrichment; ZBP1 RIP	[57]
Splicing and hybrid failure	Retained introns; mis-spliced exons; RNA–RNA duplexes; persistent RNA–DNA hybrids	RanGTP-dependent export separates nuclear production from cytosolic sensing	Immature nuclear RNA → compartment-accessible ligand	RNase H/dsRNase discrimination; SF3B perturbation; export blockade	[58]
Chromatin and repeat derepression	H3K9me3 loss; HP1 disruption; tau-associated heterochromatin collapse; ERV activation	Repeat transcription rises before Z-RNA accumulation; Zbp1 reduction rescues pathology in genetic models	Silenced repetitive loci → ligand-producing transcript reservoir	H3K9me3/HP1 profiling; ERV knockdown; Zbp1 rescue	[37]
Oxidative mitochondrial licensing	ROS; 8-oxoguanine; mtDNA fragmentation; TFAM displacement; membrane escape	Oxidation, cleavage, translocation and persistence are separable checkpoints	Compact nucleoid → accessible cytosolic Z-DNA candidate	8-oxoG mapping; mtDNA depletion; fractionation; direct structural evidence	[59]
Signal commitment and ligand confirmation	Zα-dependent occupancy; ZBP1 oligomerization; RHIM–RIPK3 assembly; MLKL activation	~87% overlap between Z-form-enriched and ZBP1-bound murine RNAs in one survey	Structural compatibility → necroptotic or PANoptotic signalling	Structure + occupancy + Zα dependence + perturbation + rescue	[60]

**Table 2:** Integrates ligand origin, signalling-complex assembly, receptor proteostasis, and execution-pathway activation to define the molecular transitions governing microglial fate and the biomarkers most informative for state-specific therapeutic intervention.

State	Ligand Context	Dominant Signalling Module	Molecular Output	Defining Readouts	Therapeutic Logic	References
Acute adaptive activation	Neuron-derived extracellular-vesicle mtDNA after mild injury	ZBP1–TBK1–IRF3	Transient IL-6/TNF induction; preserved viability	p-TBK1, p-IRF3, cytokine pulse; absent p-MLKL	Avoid indiscriminate early blockade	[100]
RIPK1-biased inflammation	Oxidised mitochondrial Z-DNA in amyloid-stressed microglia	ZBP1–RIPK1 kinase–NF-κB axis	Persistent inflammatory transcription without obligatory lysis	p-RIPK1, NF-κB targets, viable microglia; weak RIPK3–MLKL signal	Target chronic RIPK1 activity	[101]
Proteostatic amplification	Heme plus TNF-α under sustained injury	AKT–USP4–ZBP1 axis	Reduced ZBP1 ubiquitination and prolonged receptor stability	p-AKT, stabilised USP4, reduced polyubiquitinated ZBP1	Destabilise USP4 or restore receptor turnover	[90]
Execution-complex assembly	Persistent ligand with cytokine and damage-signal convergence	ZBP1–RIPK3/RIPK1–FADD–caspase-8–inflammasome network	Coupled apoptotic, necroptotic and pyroptotic signalling	RIPK3 activation, cleaved caspase-8/3, ASC assembly, caspase-1	Multinode inhibition before terminal commitment	[102,103]
Necroptotic commitment	Sustained RHIM-dependent ZBP1 signalling	RIPK3–MLKL	MLKL oligomerisation, membrane permeabilisation and lytic death	p-MLKL, MLKL membrane translocation, LDH release	Block RIPK3 or MLKL after pathway engagement	[104]
PANoptotic microglial loss	Severe traumatic injury with heme–TNF synergy	Integrated inflammasome–caspase–RIPK3 programme	Destructive inflammatory death and microglial depletion	Concurrent p-MLKL, cleaved caspases, GSDMD cleavage and inflammasome markers	Preserve the microglial pool by suppressing receptor stabilisation	[105]
Resolution versus persistence	Ligand clearance and receptor degradation versus continued mtDNA/Z-RNA exposure	Ubiquitin-dependent ZBP1 turnover versus sustained scaffold availability	Return to surveillance or chronic inflammatory reinforcement	ZBP1 half-life, ubiquitination, ligand persistence, p-RIPK1/p-MLKL ratio	Time treatment to molecular state, not ZBP1 abundance alone	[106]

**Table 3:** Comparative reconstruction of ZBP1-associated disease and experimental endotypes. The table summarizes the proposed ligand, producer–sensor relationship, dominant signalling pathway, level of mechanistic support, extent of human validation, therapeutic relevance, and supporting references for each model.

Disease/Model	Proposed Ligand	Producer → ZBP1-Sensing Cell	Main ZBP1-Associated Pathway/Output	Evidence Level	Human Validation	Therapeutic Relevance	Reference
Tauopathy/PS19	Endogenous Z-RNA	Neuron → neuron	ZBP1 → RIPK3 → MLKL → neuronal injury	Strong preclinical; genetic + biochemical	Partial; neuronal ZBP1 associated with poorer cognition in AD; direct human neuronal necroptosis unconfirmed	ZBP1/RIPK3/MLKL inhibition; experimental	[88]
Amyloid-associated pathology	Z-form-associated oxidized mtDNA	Microglia → microglia	ZBP1 → RIPK1 → inflammatory transcription	Strong preclinical; genetic + pharmacological	Limited mechanistic validation	Suppression of chronic plaque-associated inflammation; preservation of adaptive microglial functions required	[159]
Mild traumatic brain injury	EV-associated neuronal mtDNA	Injured neuron → microglia	ZBP1 → TBK1 → IRF3 → IL-6/TNF	Moderate–strong preclinical; genetic + transcellular	Limited	Therapeutic timing critical; early inhibition may suppress protective microgliosis	[160]
Severe traumatic brain injury	Endogenous ZBP1 ligand incompletely resolved; heme/TNF inflammatory context	Injured tissue → microglia	USP4–ZBP1 stabilization → inflammasome activation + apoptosis + necroptosis	Preclinical mechanistic + pharmacological	Indirect; USP4 associated with poorer clinical outcome	USP4/ZBP1 proteostasis modulation; experimental	[90]
ADAR1 Zα-domain deficiency	Immunogenic endogenous dsRNA / Z-nucleic-acid-compatible substrates	Multiple neural populations → multiple sensors	MDA5–IFN-I dominant; ZBP1/PKR activated but non-dominant	Strong causal epistasis	Human same-lesion causal chain not established	Demonstrates that ZBP1 activation alone does not justify ZBP1-directed therapy	[124]
Human ligand-induced necroptosis models	Z-form-compatible nucleic acids	Human cultured cells → same cell	ZBP1 → RIPK1 → RIPK3 → MLKL	Strong mechanistic human-cell evidence	Human cellular mechanism demonstrated; human neural disease not validated	Highlights human-specific RIPK1 dependence and adaptor compatibility	[161]
cGAS–STING bystander model	Secondary Z-nucleic acids following ADAR1 degradation	DNA-sensing cell → bystander cell	cGAS–STING → IFN/TNF → ADAR1 loss → ZBP1-dependent apoptosis	Strong experimental, non-neural	Not demonstrated in neurons	Illustrates secondary ZBP1 recruitment downstream of upstream sensor failure	[162]

## Data Availability

No new data were created or analyzed in this study. Data sharing is not applicable to this article.
